# Gene expression signatures of neuroendocrine prostate cancer and primary small cell prostatic carcinoma

**DOI:** 10.1186/s12885-017-3729-z

**Published:** 2017-11-13

**Authors:** Harrison K. Tsai, Jonathan Lehrer, Mohammed Alshalalfa, Nicholas Erho, Elai Davicioni, Tamara L. Lotan

**Affiliations:** 1 Department of Pathology, Johns Hopkins University School of Medicine, Baltimore, MD USA; 2 GenomeDx Biosciences, Vancouver, British Columbia Canada; 3 Department of Oncology, Johns Hopkins University School of Medicine, Baltimore, MD USA; 4 Present address: Department of Pathology, Brigham and Women’s Hospital, Boston, MA USA

**Keywords:** Neuroendocrine prostate cancer, Small cell carcinoma, Mixed prostatic adenocarcinoma, FFPE, Gene signature, Meta-analysis, Nearest centroid classifier

## Abstract

**Background:**

Neuroendocrine prostate cancer (NEPC) may be rising in prevalence as patients with advanced prostate cancer potentially develop resistance to contemporary anti-androgen treatment through a neuroendocrine phenotype. While prior studies comparing NEPC and prostatic adenocarcinoma have identified important candidates for targeted therapy, most have relied on few NEPC patients due to disease rarity, resulting in thousands of differentially expressed genes collectively and offering an opportunity for meta-analysis. Moreover, past studies have focused on prototypical NEPC samples with classic immunohistochemistry profiles, whereas there is increasing recognition of atypical phenotypes. In the primary setting, small cell prostatic carcinoma (SCPC) is frequently admixed with adenocarcinomas that may be clonally related, and a minority of SCPCs express markers typical of prostatic adenocarcinoma while rare cases do not express neuroendocrine markers. We derived a meta-signature of prototypical high-grade NEPC, then applied it to develop a classifier of primary SCPC incorporating disease heterogeneity.

**Methods:**

Prototypical NEPC samples from 15 patients across 6 frozen tissue microarray datasets were assessed for genes with consistent outlier expression relative to adenocarcinomas. Resulting genes were used to determine subgroups of primary SCPCs (N=16) and high-grade adenocarcinomas (N=16) profiled by exon arrays using formalin-fixed paraffin-embedded (FFPE) material from our institutional archives. A subgroup classifier was developed using differential expression for feature selection, and applied to radical prostatectomy cohorts.

**Results:**

Sixty nine and 375 genes demonstrated consistent outlier expression in at least 80% and 60% of NEPC patients, with close resemblance in expression between NEPC and small cell lung cancer. Clustering by these genes generated 3 subgroups among primary samples from our institution. Nearest centroid classification based on the predominant phenotype from each subgroup (9 prototypical SCPCs, 9 prototypical adenocarcinomas, and 4 atypical SCPCs) achieved a 4.5% error rate by leave-one-out cross-validation. The classifier identified SCPC-like expression in 40% (2/5) of mixed adenocarcinomas and 0.3-0.6% of adenocarcinomas from prospective (4/2293) and retrospective (2/355) radical prostatectomy cohorts, where both SCPC-like retrospective cases subsequently developed metastases.

**Conclusions:**

Meta-analysis generates a robust signature of prototypical high-grade NEPC, and may facilitate development of a primary SCPC classifier based on FFPE material with potential prognostic implications.

**Electronic supplementary material:**

The online version of this article (10.1186/s12885-017-3729-z) contains supplementary material, which is available to authorized users.

## Background

Neuroendocrine prostate cancer (NEPC) is a rare aggressive variant of prostate cancer comprising a spectrum of diseases emerging in different clinical settings, from de novo primary small cell prostatic carcinoma (SCPC) to treatment-related metastatic NEPC [1]. The 2016 WHO classification of NEPC consists of adenocarcinoma with neuroendocrine differentiation (Ad+NED), well-differentiated neuroendocrine tumor, small cell neuroendocrine carcinoma (synonymous with SCPC), and large cell neuroendocrine carcinoma (LCNEC), of which the last two are particularly aggressive and referred to in this paper as high-grade NEPC. Prevalence of NEPC is anticipated to rise as patients with metastatic prostate cancer receive newer anti-androgen treatments and potentially develop resistance through a neuroendocrine phenotype [2].

Molecular characteristics associated with high-grade NEPC include absence of androgen receptor (AR) signaling, RB loss combined with p53 dysfunction, and reduced REST activity together with up-regulation of neuroendocrine genes [3, 4]. Diagnosis is often supported through immunohistochemistry (IHC) of corresponding proteins, with high-grade NEPC exhibiting the prototypical profile of negative AR, high Ki-67, and positive neuroendocrine markers. In the primary setting however, IHC studies have demonstrated PSA positivity in 17-20% of SCPC and retention of other markers associated with adenocarcinomas in up to 25%, while panels of neuroendocrine markers can be entirely negative in up to 12% [5, 6]. In the metastatic setting, intermediate NEPC-like characteristics have been observed among some adenocarcinomas progressing to androgen-independence [7, 8]. Although prognostic implications of atypical features have not been formally established, rare hybrid tumors with aggressive progression have been described [9, 10].

Diagnostically, NEPC may be challenging to distinguish histologically from poorly differentiated high-grade adenocarcinoma, however prompt recognition is important since NEPC is relatively resistant to anti-androgen treatment but initially sensitive to platinum-based chemotherapy. Comparisons of NEPC and adenocarcinomas have led to candidates for diagnostic markers or targeted therapy, such as AURKA [11]. Studies have generally been based on few NEPC patients with classic immunophenotype and have resulted in at least 8 lists with thousands of differentially expressed genes collectively [4, 8, 11–15], suggesting potential opportunity for meta-analysis. Alternatively, larger populations of NEPC tumors might be profiled by leveraging archived formalin-fixed paraffin-embedded (FFPE) diagnostic samples. Improved technology has demonstrated gene expression concordance between FFPE and fresh frozen tissue despite RNA degradation in FFPE, with an ability to detect molecular subtypes of prognostic and predictive importance [16, 17].

In this study, we first compared and assessed published NEPC gene expression studies on the level of differentially expressed gene-lists, cohort details, and gene expression signatures. Using a meta-analysis approach, we consolidated common patterns of prototypical high-grade NEPC, specifically identifying genes with consistent outlier expression among SCPC and LCNEC samples of classic immunophenotype across 6 frozen tissue microarray datasets, yielding a 69-gene model with almost indistinguishable behavior between high-grade NEPC and small cell lung cancer (SCLC). We next analyzed an FFPE exon array dataset from our institution (JHU-FFPE) profiling 16 primary SCPCs and 16 adenocarcinomas (predominantly Gleason 9), notable for inclusion of mixed cases, AR-positive SCPCs, PSA-positive SCPCs, and NE-marker negative SCPCs. Based on meta-analysis genes, we identified 3 subgroups (labeled prototypical SCPC, prototypical adenocarcinoma, and atypical SCPC) and developed a LIMMA-based 3-centroid-classifier. Although we lacked a validation set, the classifier achieved a 4.5% estimated error rate on leave-one-out cross-validation and detected SCPC-expression in 40% (2/5) of mixed adenocarcinomas and 0.3-0.6% of adenocarcinomas from radical prostatectomy (RP) cohorts, with a possible enrichment for adverse events.

## Methods

### NEPC gene-lists in the literature

We searched the literature for published gene-lists of differentially expressed genes between NEPC and prostatic adenocarcinoma based on expression profiling of patient tumor samples or patient-derived xenografts (Table 1) [4, 8, 11–15]. To compare gene-lists and identify common genes, we updated gene names and probe assignments with current HGNC symbols, and where possible, resolved un-annotated probes and non-standard transcripts through BLAT alignment of underlying sequences to hg19. For rough statistical assessment of similarity, we evaluated pair-wise overlaps of gene-sets via Fisher exact test, with a presumptive background of ~20000 genes.

**Table 1:** Differential expression studies between NEPC & prostatic adenocarcinoma

		NEPC samples				AdCa samples					Gene-list	
Study	Type	U	M	L	P	U	M	L	P	GS	Up	Dn
WCMC mCRPC	T	10	10	3	2	25	18	9	7		1226	1132
WCMC 2011	T/X^a^	7	6		1	30			30	5-9	494	460
UW mCRPC	T	6	7	3		41	37	24			126	29
LuCaP xeno	X	3	2	1	1	16	5	11	4		17	16
MDA xeno	X	3		4	1	3		1	2	9	45	67
VPC xeno	X	1			1	6		1	11		254	185
VPC 2012	T/X*	1	3			3		2	3	7-9	202	127
JHU 2009	T	1			1	1			1	7	41	41
Total		29				114					1782	1785

Legend: (Notation): U: unique patients, T: tumor, X: xenograft, P: primary, M: metastases, L: bladder, rectum, or lymph node, GS: Gleason score, Up/Dn: size of gene-lists up/down-expressed in NEPC. (^a^): WCMC 2011 and VPC 2012 each contained one xenograft NEPC sample. (Notes): WCMC studies shared 2 NEPC patients, while UW mCRPC and LuCaP studies shared 1 NEPC patient. Adenocarcinomas with NE differentiation were grouped with NEPCs in WCMC mCRPC, with adenocarcinomas in MDA xeno, and with either cohort in UW mCRPC depending on IHC status of chromogranin and synaptophysin (NEPC when both positive). The VPC xeno gene-list cross-referenced other studies and consisted of genes with expression changes following transdifferentiation in a xenograft model, concomitant alterations in the same direction prior to transdifferentiation relative to adenocarcinomas, and exhibiting the same trend in WCMC 2011. The JHU study consisted of a single patient tumor with adjacent small cell carcinoma and adenocarcinoma components

### Bioinformatic processing and analysis

We collected various datasets for meta-analysis and ancillary tests (Table 2). Microarrays were processed by RMA-based pipelines to arrive at absolute log-intensities. Gene signatures of AR signaling (ARS) (“Hieronymus up” genes) [18], neuronal phenotype (Lapuk) [4], and cell cycle progression (CCP) (Cuzick) [19] were scored by average expression. LIMMA and DAVID/PANTHER were used for differential expression and gene-ontology analyses [20]. Details are provided in Additional file 1.

**Table 2:** Gene expression datasets used

Dataset	Platform	Source	Patients		
			NEPC	AdCa	Ad+NED
Meta-analysis NEPC datasets					
LuCaP xeno	Agilent	Internal	3	16	
VPC xeno	Agilent	GSE41192	2	6	
MDA xeno	Affymetrix 3'	GSE32967	3	2	1
MDA l-CRPC	Agilent	GSE33277	4	16	
UM mCRPC	Agilent	GSE35988	2	33	
UW mCRPC	Agilent	GSE66187	2	41	4
Other NEPC and prostate datasets					
SU2C (mCRPC)	RNA-seq	cBioPortal	5^a^	113	*
WCMC (mCRPC)	RNA-seq	cBioPortal	10^a^	25	*
TCGA	RNA-seq	cBioPortal		333	
JHU-FFPE	Affymetrix Exon	GSE104786	16	16	1
Mayo-FFPE	Affymetrix Exon	GSE61126		235	
MSKCC	Affymetrix Exon	GSE21034		150	
UW-extra	Agilent	GSE77930	2	39	2
GRID datasets (FFPE)					
Prospective	Affymetrix Exon	GenomeDx		2293	
JHU-RP	Affymetrix Exon	GSE79958		355	
Mayo	Affymetrix Exon	GSE46691		780	
		GSE61126			

Legend: ^a^NEPCs in SU2C and WCMC included adenocarcinomas with NE differentiation, SCPCs, and LCNECs, but without specification of subtype. NEPCs in other datasets were entirely SCPCs except for one LCNEC sample from MDA xeno

### Outlier-based meta-analysis

For an NEPC sample, a gene was considered an outlier if its expression was greater than 2 standard deviations and log2-fold change 1 away from the mean of the dataset’s adenocarcinoma cohort. For adenocarcinomas, this definition was applied after first removing the evaluated sample from the adenocarcinoma cohort, although not possible for the smallest dataset. For each gene, the number of NEPC (or adenocarcinoma) samples with outlier up-expression or down-expression was tabulated and further summarized by patient using fractional counts for multiple samples from the same patient. Genes with outlier status in the same direction in N or more NEPC patients were referred to as meta-N genes. NEPC and adenocarcinoma centroids were similarly calculated on the patient level through fractional weights, and used for correlation-based scoring and classification.

### JHU-FFPE patient sample selection

Thirty-three FFPE samples (Table 3), diagnosed as 16 SCPC’s, 16 high-grade adenocarcinomas (majority Gleason 9), and 1 adenocarcinoma with neuroendocrine differentiation, including 4 matched pairs from mixed tumors, were retrieved from surgical pathology and consultation files of Johns Hopkins Hospital from 1999-2013 after IRB approval and successfully processed for gene expression profiling with Human Exon 1.0 ST GeneChips (Affymetrix), as described in a previous study using 22 of these samples [21]. Diagnoses were in accordance with recently proposed morphologic criteria of neuroendocrine differentiation in prostate cancer [1]. A tissue microarray (TMA) containing 11 of the 33 samples with IHC of Rb1 and cyclin D1 was described previously [21], and additional IHC was performed for the prostate-related markers PSA (Ventana), AR (Ventana SP107), and Nkx3.1 (Biocare), and the neuroendocrine markers chromogranin A (Ventana LK2H10), synaptophysin (Novocastra 27G12), and CD56 (Cell Marque 123C3.D5) [1, 22].

**Table 3:** Pathology ofPathology of JHU-FFPE dataset samples

SCPC	AdCa	Ad+NED	Gleason	Block age	Source	Type
Mixed samples (by ID)						
56104_S	56104_A		3+4	2.5	TURP	Consult
56105_S	56105_A		5+4	2.5	TURP	Consult
56321_S	56321_A		5+4	14.2	TURP	Consult
57912_S	57912_A		5+4	0.2	RP	JH
56111_S			5+4	4.2	TURP	Consult
56106			5+5	2.2	TURP	Consult
56322			4+4	1.5	TURP	Consult
57914			5+5	4.6	Biopsy	Consult
57916			5+4	3.7	Biopsy	Consult
57917			5+4	4.6	Biopsy	Consult
	57918_A		5+4	3.8	Biopsy	Consult
Small cell only samples						
54674				15.9	Autopsy	JH
56057				5.7	Biopsy	JH
56107				0.9	TURP	Consult
56110				2.2	TURP	Consult
57915				2.3	Biopsy	Consult
57920				2.9	Biopsy	Consult
Adenocarcinoma only samples						
	57585		4+5	2.0	Biopsy	JH
	57589		4+5	2.0	Biopsy	JH
	57591		4+5	1.9	Biopsy	JH
	57619		4+5	2.6	Biopsy	JH
	57632		4+5	2.5	Biopsy	JH
	57634		5+5	2.4	Biopsy	JH
	57637		4+5	2.3	Biopsy	JH
	57640		4+5	2.1	Biopsy	JH
	57641		5+4	2.1	Biopsy	JH
	57642		4+5	2.0	Biopsy	JH
Adenocarcinomas with NE differentiation						
	56061_A	56061_S	5+4	3.7	Biopsy	JH

### LIMMA-based centroid models

For binary classification based on training subgroups A and B, LIMMA was used for feature selection (differentially expressed genes between A and B with adjusted *p*-values < 0.05), and a nearest centroid model based on A and B was developed. For ternary classification based on training subgroups A, B, and C, feature selection consisted of differentially expressed genes common to 2 or more LIMMA comparisons (A versus B, A versus C, and B versus C), and a nearest centroid model based on A, B, and C was developed. Leave-one-out cross-validation (LOOCV) with mixed pairs removed together was used to evaluate models, starting from new feature selection upon each removal.

### GRID**®** database

Expression profiles (N=3428) of adenocarcinomas from RP specimens were retrieved from Decipher GRID**®** prostate cancer database [23], consisting of high risk cases from clinical use of the Decipher test (NCT02609269; Prospective cohort) or from retrospective institutional studies with outcomes data (JHU-RP and Mayo cohorts) [24–26]. Specimen selection, RNA extraction, and Human Exon 1.0 ST Array hybridization were done in a Clinical Laboratory Improvement Amendments (CLIA/CAP/NYS)-certified laboratory facility (GenomeDx Biosciences, San Diego, CA, USA) as previously described [27]. Normalization was performed using Single Channel Array Normalization (SCAN).

## Results

### Literature NEPC gene-lists comprise thousands of genes with significant overlap but no universal genes despite a common NEPC immunophenotype and common gene signature patterns

We identified 8 gene-lists from the literature comparing gene expression of NEPCs and prostatic adenocarcinomas, based on a collective total of 29 and 114 unique patients respectively (Table 1) [4, 8, 11–15]. Cohort definitions varied slightly between studies, specifically regarding treatment of adenocarcinomas with NE differentiation, which were grouped with NEPCs in WCMC mCRPC, with adenocarcinomas in MDA xeno, and variably with either cohort depending on IHC status in UW mCRPC (grouped with NEPC when synaptophysin and chromogranin both positive). NEPC cohorts thus contained significant proportions of adenocarcinomas with NE differentiation for WCMC mCRPC and UW mCRPC (46% and 50% of NEPCs respectively), but otherwise consisted exclusively of SCPCs and one rare LCNEC for most gene-lists (6 of 8). IHC of annotated SCPCs and the LCNEC, when provided, was always negative for PSA (17/17 patients) and AR (10/10), always positive for synaptophysin (17/17), and usually positive for chromogranin (9/15). Thus most gene-lists, in particular the 6 of 8 based on SCPCs / LCNEC, corresponded to a classic NEPC immunophenotype and notably lacked AR-positive or PSA-positive SCPCs, which have been reported in 17-20% of primary SCPCs [5, 6].

Collectively, the 8 gene-lists consisted of 1782 up-genes and 1785 down-genes with increased and decreased expression in NEPC, including 433 (24%) and 235 (13%) common to multiple lists although some studies were not entirely independent (Additional file 2: Table S1). No genes were common to all lists, with the most frequent comprised of 9 largely neuronal up-genes in 5/8 lists (BSN, CRMP1, GPRIN1, INA, MAST1, MYT1, RAB3C, SNAP25, UNC13A) and 5 largely androgen-related down-genes in 4/8 lists (CYP1B1, KLK2, KLK3, STEAP1, TRPV6). Gene-lists demonstrated pair-wise similarity, often related to cohort or statistical details (Additional file 2: Table S**2**); the study with greatest statistical power (WCMC mCRPC) generated the largest list (>2000 genes) [8] and overlapped most with other gene-lists, while comparisons of metastatic NEPC versus primary adenocarcinoma (WCMC 2011, VPC 2012) resulted in enrichment of metastasis-associated genes (Additional file 2: Table S**3**).

**Fig. 1:**
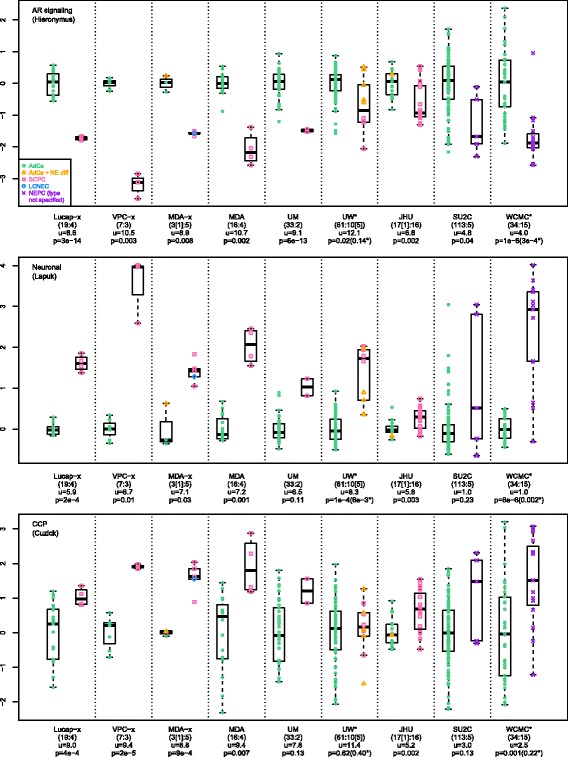
Gene signature scores across datasets profiling NEPC and adenocarcinomas. (A[A’]:B[B’]) denotes cohort sizes of A adenocarcinomas and B NEPCs including A’ or B’ adenocarcinomas with NE differentiation, u denotes mean score of the adenocarcinoma cohort, p denotes *p*-value under t-test comparison of NEPCs versus adenocarcinomas, and (*) signifies p-values after averaging over multiple samples from the same patient. ARS and neuronal phenotype scores completely separated cohorts (AUC 100%) in xenograft and frozen tissue primary datasets (Lucap-x, VPC-x, MDA-x, MDA), and ARS demonstrated significant cohort differences (*p*<0.05) across all datasets. CCP was highly correlated to an RB loss signature (mean r=0.96 across datasets; not shown), in agreement with reports showing correlation of CCP and E2F1 targets [7]. In UW, NEPCs annotated as adenocarcinomas with NE differentiation mostly demonstrated ARS and CCP scores similar to adenocarcinomas. In WCMC and SU2C, NEPCs also sometimes demonstrated gene signature scores similar to adenocarcinomas, and may have corresponded to adenocarcinomas with NE differentiation, however NEPC subtypes were not specified in annotations provided. In JHU-FFPE, 5 SCPCs exhibited ARS scores similar to adenocarcinomas (fold-change > -0.5 and z-score > -1), and are investigated further in the JHU-FPE results section. JHU-FFPE scores also demonstrated the least dynamic range across gene signatures, likely related to RNA degradation in FFPE. Gene signature scores were formed by average expression of genes. Among single-sample scoring methods, SVD-based PLAGE has been recognized as a top performer and is equivalent to (signed) average expression for perfectly correlated (and anti-correlated) genes. Indeed, PLAGE and average expression were highly correlated across the NEPC datasets (correlations for CCP > 0.99, Neuronal > 0.96, ARS > 0.95)

We obtained available NEPC gene expression data corresponding to 5 of the 8 gene-lists, 3 more studies with known SCPCs (including an FFPE dataset from our institution), and 1 study (SU2C) with rare NEPCs consisting mostly (80%) of adenocarcinomas with NE differentiation (Table 2). Gene signature scores were used to assess samples (Fig. 1), similar to a recent study [7]. Annotated SCPCs (and the LCNEC) from frozen tissue datasets almost always demonstrated a prototypical pattern of low ARS, high neuronal phenotype, and high CCP scores, in accordance with a classic NEPC immunophenotype. In xenograft and frozen tissue primary datasets, ARS and neuronal phenotype scores completely separated SCPCs / LCNEC from adenocarcinomas (AUC 100%). Annotated adenocarcinomas with NE differentiation generally demonstrated gene signature scores similar to adenocarcinomas, except possibly with slightly elevated neuronal phenotype scores. A few NEPCs from WCMC and SU2C also demonstrated gene signature scores similar to adenocarcinomas, and possibly represented adenocarcinomas with NE differentiation, however specific NEPC subtype was not provided in annotations of these datasets [8].

### Outlier-based meta-analysis identifies NEPC expression patterns on the patient level

We produced a meta-analysis signature of prototypical high-grade NEPC (omitting adenocarcinomas with NE differentiation) by utilizing 6 frozen tissue microarray datasets profiling 23 NEPC samples (from 15 patients) with SCPC or LCNEC morphology, classic immunophenotype (when provided), and low ARS and high neuronal phenotype scores (Table 2 **,** Additional file 2: Table S4) [12–14, 21, 28, 29]. These datasets largely contained NEPCs and adenocarcinomas from similar clinical stages, ideally reducing confounding effects; known adenocarcinomas with NE differentiation were considered separately. RNA-seq datasets were excluded from meta-analysis as it was not possible to separate adenocarcinomas with NE differentiation from the NEPC cohorts based on available annotations. The FFPE dataset, which will be analyzed in detail in a later section, was excluded due to attenuated expression and cohort heterogeneity. We compiled the meta-12 (Table 4) and meta-9 (Additional file 2: Table S5) gene-sets, comprised of 69 and 375 genes with consistent outlier status in at least 80% (12/15) and 60% (9/15) of high-grade NEPC patients. Meta-12 genes, which required agreement between NEPCs from at least 4 datasets due to cohort sizes, were enriched for “generation of neurons” (adj p=2.6e-6 in up-genes) and “androgen receptor signaling” (adj p=3.8e-3 in down-genes) but not cell cycle. Rather, “cell division” became the most enriched gene-ontology term among meta-9 up-genes (adj p=2.6e-6), partly due to cell-cycle genes meeting outlier criteria in primary but not necessarily metastatic NEPC. Most meta genes appeared in the literature: 90% of meta-12 including AR, ASCL1, SRRM4, and CCND1, and 78% of meta-9 including PEG10, REST, EZH2, CHGA, and RB1, as expected since published NEPC gene-lists (Additional file 2: Table S1) used 9 of the NEPC patients. However, outlier analysis potentially missed genes with modest fold-changes or large variability such as HIST1H4C, which was an outlier in 55% of NEPC patients but increased to 92% under relaxed criteria. Metastatic CRPC NEPC samples demonstrated the least outlier agreement overall, while rare adenocarcinomas had NEPC-like outlier behavior and were often associated with notable features (Additional file 3: Figure S1).

**Table 4:** Meta-12 genes with outlier expression in > 80% (12/15) high-grade NEPC patients

Gene	NEPC outliers	% NEPC [15]	% AdCa [114]	% Ad+NED [5]	NEPC centroid	AdCa centroid
Up genes						
AP3B2	15	100	6	80	8.5	6.5
TUBB2B	14.4	96	4	0	11.8	7.3
CRMP1	14	93	4	60	9.9	6.7
PCSK1	14	93	3	20	10.8	6.2
SEZ6	14	93	4	60	8.5	6.5
CDC25B	13.8	92	7	0	11.5	9.5
KCNC1	13.8	92	2	40	7.8	5.7
TMEM145	13.7	91	6	40	11.7	9.1
CCDC88A	13.4	89	4	40	9.0	7.6
ASCL1	13.3	89	5	0	10.9	6.8
ENO2	13.2	88	5	0	11.6	9.4
MIAT	13.1	87	6	0	10.1	7.1
SRRM4	13.1	87	5	40	8.8	6.4
NPTX1	13	87	4	0	10.7	7.2
PHF19	13	87	6	0	10.8	9.2
RNF183	13	87	6	0	9.4	7.2
TOX	13	87	4	40	8.6	6.2
INSM1	12.9	86	4	0	10.1	6.9
IGFBPL1	12.8	85	6	0	9.5	7.1
ELAVL3	12.6	84	2	60	7.7	6.0
RUNDC3A	12.6	84	5	20	9.1	7.0
NKX2-1	12.5	83	5	0	10.0	7.8
UNC13A	12.5	83	6	100	7.8	6.0
FANCL	12.4	83	1	0	12.3	11.6
SH3GL2	12.4	83	4	20	10.0	7.0
FAM161A	12.1	81	6	0	9.6	8.9
APLP1	12	80	4	40	9.3	7.2
DLL3	12	80	4	40	9.2	6.7
DNMT1	12	80	4	20	10.5	8.7
ELAVL4	12	80	3	50	7.7	5.7
FGF9	12	80	6	0	9.5	7.3
INA	12	80	8	20	11.0	7.6
NPPA	12	80	4	0	8.6	5.9
PCSK2	12	80	1	0	8.1	5.5
SNAP25	12	80	8	100	9.5	6.2
SOX2	12	80	5	0	11.0	7.3
STMN1	12	80	3	0	10.6	9.9
Down genes						
AR	14.3	95	5	0	7.0	13.3
AIM1	14.3	95	5	20	7.7	10.2
ADRB2	14	93	3	20	7.8	11.5
SPDEF	14	93	4	0	8.9	12.0
STEAP1	14	93	5	0	7.0	12.7
STEAP2	14	93	6	0	7.6	13.3
C1orf116	13.9	93	4	0	7.8	11.4
ERGIC1	13.9	93	5	0	8.2	10.5
LATS2	13.8	92	4	0	7.3	10.5
NKX3-1	13.6	91	6	0	8.9	14.9
PMEPA1	13.6	91	4	0	10.2	14.5
HOMER2	13.4	89	3	0	7.8	10.9
ZBTB16	13.4	89	5	0	8.3	12.4
ZG16B	13.4	89	4	0	8.2	13.0
EPHX2	13.1	87	2	0	9.1	12.5
SLC45A3	13	87	5	0	9.1	14.6
GLUD1	12.7	85	4	0	10.0	12.7
SLC44A4	12.7	85	5	20	7.5	10.5
CCND1	12.6	84	8	40	6.9	10.1
KLK3	12.6	84	4	0	9.3	12.8
PPAP2A	12.6	84	7	0	10.7	14.8
GRTP1	12.5	83	4	0	6.0	7.9
YAP1	12.5	83	4	60	6.4	9.1
SYNGR2	12.4	83	4	0	10.8	13.0
ALDH6A1	12.3	82	2	0	8.7	11.7
NAP1L2	12.3	82	5	0	6.3	9.8
HPN	12.2	81	5	20	8.0	11.3
RGS10	12.1	81	4	0	10.1	14.1
RILPL2	12.1	81	4	0	9.1	11.4
ACPP	12	80	3	20	7.3	11.5
HOXB13	12	80	6	0	10.4	14.2
ICAM3	12	80	6	0	8.7	11.0

Legend: List of meta-12 genes with % outlier status [# patients] among meta-analysis patients (NEPC, adenocarcinoma, or adenocarcinoma with NE differentiation). Centroids were formed by averaging each gene over NEPC or adenocarcinoma patients

We next examined genes not present on all microarrays but still demonstrating consistent outlier expression. The most prevalent was CCEPR, overexpressed in 11.5/13 (88%) NEPC patients [30]. This sparsely studied long non-coding RNA did not appear in probe annotation files or GENCODE (v25), but was targeted by probes A_32_P216820 (Agilent), 228679_at (Affymetrix), and 3290641 (Affymetrix exon) based on BLAT; one NEPC gene-list included 228679_at without gene annotation [13]. Genomic location of CCEPR almost overlapped with the meta-9 up-gene PHYHIPL from the opposite strand, and these genes were highly correlated in meta-analysis datasets (r=0.70-0.93). PHYHIPL probe-set 226623_at moreover had the top co-expression similarity score (3.2e-138) to CCEPR probe-set 228679_at under Multi-Experiment Matrix analysis based on hundreds of Affymetrix datasets [31].

**Fig. 2:**
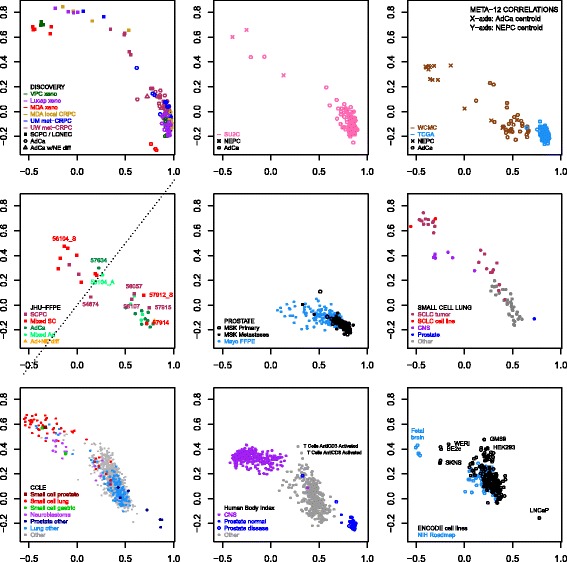
Correlation profiles relative to meta-12 adenocarcinoma and NEPC centroids across datasets. Nearest centroid classification of NEPC datasets demonstrated NEPC sensitivities and specificities of 91% and 100% on training samples, 60% and 98% in SU2C, 80% and 100% in WCMC, and 63% and 94% in JHU-FFPE. Centroid correlation profiles were also evaluated for prostatic adenocarcinoma datasets (TCGA, MSK, Mayo-FFPE) and various human tissue or cell line datasets including SCLC (GSE43346), CCLE (cBioPortal), Human Body Index (GSD7307), ENCODE (GSE19090), and NIH Roadmap (GSE18927). Correlations were generally weaker in FFPE datasets (JHU-FFPE, Mayo-FFPE) and in WCMC derived primarily from biopsies. Rare outlier adenocarcinomas were present across datasets, usually related to low ARS. SCLCs generally had the most similar centroid profile to NEPC followed by small cell gastric carcinoma and CNS-related samples. In JHU-FFPE, 5 SCPCs appeared to cluster with adenocarcinomas, demonstrated ARS scores similar to adenocarcinomas (Fig. 1, Additional file 3: Figure S3), and are discussed further in the JHU-FFPE results section

Meta-12 genes were derived from conceptually similar criteria underlying the recent integrated NEPC classifier [8]. We adopted further modifications, including nearest centroid scoring and equal weighting of patients, whereas the integrated classifier relied on a single centroid (NEPC) and utilized equal weighting of samples, with significant influence from one patient providing almost half of NEPC samples (6/13) with highly similar expression profiles. The classifiers were similarly sized (69 versus 70 genes; 11 shared), highly correlated across NEPC mCRPC datasets (UM 0.73, SU2C 0.87, WCMC 0.90), and produced identical classifications of SU2C, but disagreed on rare respective discovery samples (2 WCMC NEPCs and 2 UM adenocarcinomas). Both classifiers were based on NEPCs with below average ARS scores (WCMC initially included one NEPC with elevated ARS, which was excluded before derivation of the final classifier). Nearest centroid classification relative to meta-12 centroids (Table 4) yielded sensitivities and specificities of 91% and 100% on training samples (AUC 100% for correlation difference), and 60-80% and 94-100% in non-training NEPC datasets (Additional file 3: Figure S**2**). In non-prostate datasets, SCLC had the most similar profiles to NEPC, followed by CNS samples (Fig. 2); rare cell lines from other sites, including gastric small cell carcinomas, also resembled NEPC. In JHU-FFPE, meta-12 centroid profiles appeared to generate two main clusters, with the predominantly adenocarcinoma cluster containing 5 SCPCs. These SCPCs will be further characterized in the next section.

### JHU-FFPE demonstrates heterogeneity of primary SCPC with associated gene expression patterns relative to signatures and meta-9 genes

We used exon arrays to profile FFPE material of 16 primary SCPCs, 16 high-grade adenocarcinomas, and 1 adenocarcinoma with NE differentiation from our institutional archives (JHU-FFPE) (Table 3), intended to represent the natural heterogeneity of primary SCPC. Primary SCPC is known to frequently co-occur with adenocarcinoma (43% in the largest published series), typically of high Gleason grade (> 8 in 85% of cases) [6]. In JHU-FFPE, 10/16 (62.5%) SCPCs were mixed with adenocarcinomas, mostly of primary Gleason pattern 5 (80%), although only 4 fully matched pairs were available for gene expression profiling. Overall, JHU-FFPE adenocarcinomas were predominantly Gleason grade 9 (88%) by design, and most had primary Gleason pattern 4 (56%).

**Fig. 3:**
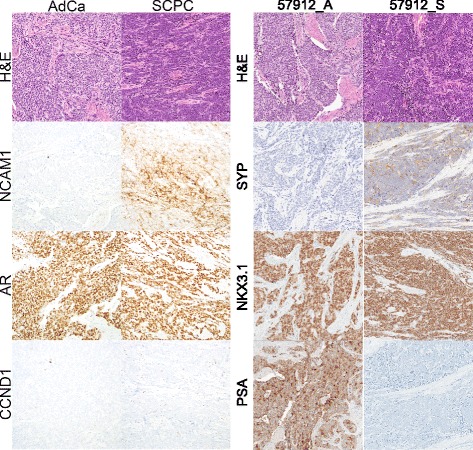
Hybrid immunohistochemistry of an unusual mixed tumor. A hybrid IHC profile was observed in an unusual mixed case from JHU-FFPE with concurrent small cell (57912_S) and Gleason 5+4 adenocarcinoma (57912_A) components. The SCPC component appeared to uniformly co-express androgen-related markers (Nkx3.1, AR) and neuroendocrine markers (synaptophysin and CD56/NCAM1 but not chromogranin) by IHC. Unusually, IHC was negative for PSA despite moderate expression of the underlying gene KLK3 (Additional file 3: Figure S11). By contrast, the adenocarcinoma component was IHC positive for PSA and negative for synaptophysin and CD56. Both components were IHC negative for cyclin D1, a proposed marker of SCPC [21]

Primary SCPC is also known to infrequently retain expression of adenocarcinoma markers (AR 17%; PSA 17-19%) or lack expression across neuroendocrine panels (12%) [5, 6]. Among SCPC samples from JHU-FFPE with available IHC status, 2/9 (22%) expressed AR robustly, 3/9 (33%) expressed AR weakly, 1/12 (9%) expressed PSA, and 1/9 (11%) had joint negativity of synaptophysin, chromogranin, and CD56 (Table 5). SCPCs with robust AR IHC (mixed 57912_S and pure 56107) exhibited unusual hybrid IHC profiles with uniform positivity of some androgen-related (AR, Nkx3.1) and neuroendocrine (synaptophysin, CD56) markers, and negativity of others (PSA and chromogranin) (Fig. 3). On the gene expression level, ARS scores were retained at levels similar to adenocarcinomas (fold-change > -0.5 and z-score > -1 relative to adenocarcinomas) in 5/16 (31%) SCPCs (Fig. 1), corresponding to the SCPCs clustering with adenocarcinomas in the meta-12 centroid profiles (Fig. 2), including both pure and mixed cases, and comprised of the SCPCs with robustly positive AR IHC (57912_S, 56107) and SCPCs with unknown AR status (56057, 57914, 57915). The robust AR-positive SCPCs both had elevated KLK3 expression despite absence of the PSA protein product on IHC. In other public datasets, annotated SCPCs with similarly retained ARS scores were rare, if present at all (Additional file 3: Figure S**3**).

**Table 5:** IHC results of selected JHU-FFPE samples

SCPC	AdCa	Rb1	ccnd1	PSA	nkx3.1	AR	chga	syp	CD56
Mixed samples (by ID)									
56104_S	56104_A	n | w	n | p	n | p	n | p	w | p	p | n	p | n	p | n
56105_S	56105_A	n | w	n | p	n | w	n | p	n | p	w | n	w | n	w | w
56321_S	56321_A	n | w	n | p	n | p	n | p	n | p	n | n	w | n	w | n
57912_S	57912_A		n | n	n | p	p | p	p | p	n | n	p | n	p | p
56111_S		n	n	n	n	w	w	p	p
56106		n	n	n	n	n	n	w	w
56322		n	p	n	n	w	n	n	n
Small cell only samples									
56107		p	n	n	w	p	n	p	n
56110		n	n	n	n	n	n	p	p

Legend: IHC data was available from a tissue microarray including 11 of the samples and for the radical prostatectomy mixed case (57912_A and 57912_S), and scored as positive (p), negative (n), or weak (w). Chromogranin A status for 57912_A and 57912_S was obtained from the diagnostic report

**Fig. 4:**
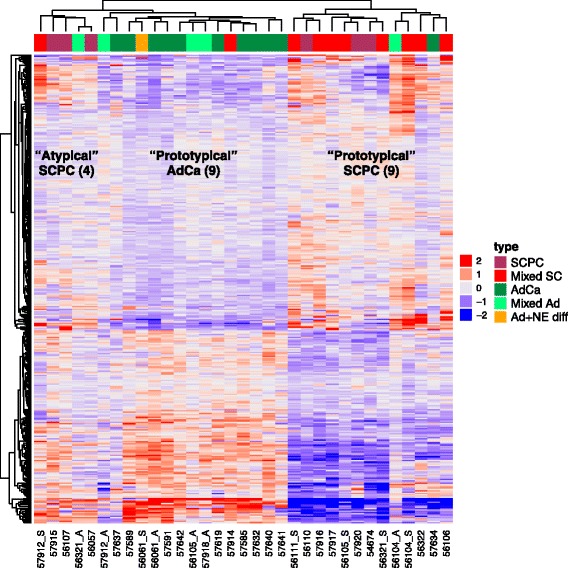
Hierarchical clustering of JHU-FFPE relative to meta-9 genes. There were 3 main groups, which we labeled “prototypical” adenocarcinomas, “prototypical” SCPCs, and “atypical” SCPCs, and which generally corresponded to pure adenocarcinomas, SCPCs with reduced ARS, and SCPCs with retained ARS respectively. The only exceptions were one SCPC outlier with retained ARS (57914) that clustered with prototypical adenocarcinomas, one pure adenocarcinoma outlier (57634) described previously in a case report that clustered with prototypical SCPCs, and heterogeneous behavior of mixed adenocarcinomas. The adenocarcinoma 57634 clustered near 56322, an SCPC with negative IHC for all 3 neuroendocrine markers synaptophysin, chromogranin, and CD56. The oldest SCPC’s (54674 and 56321_S) had low CCP and also clustered together. Meta-9 clustering was consistent with subsequent nearest centroid classification based on 9 prototypical SCPC, 9 prototypical adenocarcinoma, and 4 atypical SCPC with LIMMA-based feature selection (Fig. 6)

Hierarchical clustering relative to meta-9 genes generated 3 main subgroups, labeled “prototypical” adenocarcinomas, “prototypical” SCPCs, and “atypical” SCPCs, which generally corresponded to pure adenocarcinomas, SCPCs with reduced ARS, and SCPCs with retained ARS respectively (Fig. 4). The exceptions were one SCPC outlier with retained ARS (57914) that clustered with prototypical adenocarcinomas, one pure adenocarcinoma outlier (57634) described previously in a case report for its unusually aggressive clinical progression [32] that clustered with prototypical SCPCs, and heterogeneous behavior of mixed adenocarcinomas. Highly similar hierarchical clusters were generated using the collective genes of the ARS, CCP, and neuronal phenotype signatures, of which 38% (49/128 genes) overlapped with meta-9 genes. By contrast, hierarchical clustering relative to meta-12 genes (noted previously to lack enrichment for cell cycle) failed to produce the subgroup of SCPCs with retained ARS.

**Fig. 5:**
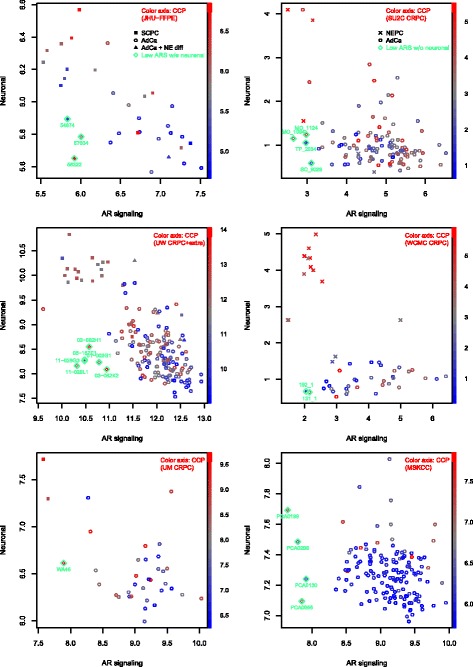
Low ARS without elevated neuronal phenotype samples across clinical datasets. Twenty samples with low ARS and low/average neuronal phenotype scores were identified based on outlier-style cut-offs relative to adenocarcinomas (fold-change < -1, z-score < -2 for ARS; fold-change < 0.5, z-score < 1 for neuronal phenotype), including known unusual cases such as the case report adenocarcinoma 57634 (JHU-FFPE), and also samples from pure adenocarcinoma datasets (MSKCC). Differential expression analysis was notable for down-expression of RAB3B in this group relative to the remaining adenocarcinomas or NEPCs (Additional file 3: Figure S4). These samples also demonstrated a wide range of CCP scores (color axis), where low CCP possibly reflected response to treatment (Additional file 3: Figure S5)

The pure adenocarcinoma outlier (57634), which behaved similar to prototypical SCPCs under meta-9 and also meta-12, clustered adjacent to the SCPC with joint neuroendocrine marker negativity (56322). Both samples were characterized by low ARS, non-elevated neuronal phenotype, and high CCP scores relative to adenocarcinomas (Fig. 5). We queried for the first 2 joint conditions in other datasets (relaxing the CCP constraint initially), specifically searching for outlier ARS scores (fold-change < -1, z-score < -2) and non-elevated neuronal phenotype scores (fold-change < 0.5, z-score < 1), with slightly relaxed ARS criteria (fold-change < -0.75, z-score < -1.5) for JHU-FFPE and WCMC CRPC due to attenuated expression. We identified 20 such clinical samples from 18 patients across metastatic datasets (Fig. 5). RAB3B, up-regulated in prostate cancer through AR [33], was the top-most jointly differentially expressed gene in this subgroup, with reduced expression relative to either NEPCs or adenocarcinomas (Additional file 3: Figure S**4**). CCP levels varied widely among these samples. High levels occurred across multiple datasets and included UM WA46, which was noted to have morphologic features of prostate cancer with NE differentiation [8]. Low levels potentially reflected response to treatment, as demonstrated in a previous study where ARS and CCP decreased in every patient after ADT (Additional file 3: Figure S5) [34]. This variation in CCP may partially explain the discordance between a recent report of negative correlation between AR signaling and proliferation signatures in metastatic CRPC versus earlier analysis reporting positive correlation between AR and E2F1 [7, 35].

Mixed adenocarcinomas were distributed among all 3 meta-9 clustering subgroups, possibly associated with degree of clonal relation with SCPCs. Clonal genomic alterations shared by components of a mixed tumor have been observed in key SCPC genes such as TP53 [15], and are capable of driving gene expression changes despite maintenance of morphology; for instance, gene expression changes intermediate to SCPC were recently reported in a xenograft model of transdifferentiation derived from a primary prostatic adenocarcinoma with bi-allelic alterations in TP53, RB1, and PTEN [12, 36]. On the other hand, mixed tumors are also susceptible to improper sampling, especially when components are intermingled. One mixed adenocarcinoma (56104_A), which clustered adjacent to its SCPC component (56104_S), was suspicious for such contamination. It unusually had the highest CCP score among JHU-FFPE adenocarcinomas (and #6 overall versus #2 for 56104_S) despite having the lowest Gleason grade (3+4), and one of the highest neuroendocrine phenotype scores (#3 overall versus #1 for 56104_S), including elevated expression levels of genes underlying chromogranin, synaptophysin, and CD56 despite IHC negativity. On one TMA core of the mixed tumor, an adenocarcinoma gland appeared upon deeper cuts of the SCPC component, demonstrating their close proximity (Additional file 3: Figure S6). We also speculated whether the mixed SCPC outlier (57914) might similarly be contaminated with adenocarcinoma, but had no evidence other than the remote possibility gleaned from its diagnostic report, which noted areas of merging with Gleason grade 5+5 prostatic adenocarcinoma.

### Meta-9 derived subgroups yield a differential expression based classifier for prototypical and atypical SCPC in the primary setting

Comparison of SCPC and adenocarcinomas from JHU-FFPE produced 385 differentially expressed genes by LIMMA (111 up, 274 down) (Additional file 3: Figure S6), including 124 (32%) from literature NEPC gene lists. Down-genes included numerous prostate specific genes (e.g., KLK3, NKX3-1) and the known NEPC-related genes CCND1 and REST [4]. Up-genes were enriched for “cell cycle” (adj p=7.8e-10) but included only 1 neuronal phenotype gene despite presence of the neuronal gene repressor REST among the down genes. We explored the exon array’s ability to detect known truncated splice variants associated with reduced REST activity, given that probe-set 2728423 targeted the 50-62bp cryptic exon found in neuroblastoma (hREST-N62), small cell lung cancer (sREST), and presumably NEPC [14, 37, 38]. There was no evidence of cryptic exon use in JHU-FFPE, however we could not rule out poor probe-set performance (Additional file 3: Figure S7) [39]. Differential expression increased substantially by reducing cohort heterogeneity (e.g., 5.8-fold to 2235 genes by removing SCPCs with retained ARS). Nearest centroid classification, based on SCPC versus adenocarcinoma with LIMMA feature selection, reflected this known heterogeneity and achieved an estimated error rate of 25% (8/32) under LOOCV, with incorrect predictions of cases highlighted by meta-9 clustering: the 5 SCPCs with retained ARS, the 2 mixed adenocarcinomas clustering with SCPCs, and the pure adenocarcinoma outlier.

We constructed a new set of cohorts based on meta-9 clusters. We selected 9 prototypical SCPCs and 9 prototypical adenocarcinomas by excluding non-standard samples: specifically mixed adenocarcinomas, the outlier adenocarcinoma, adenocarcinomas associated with NE differentiation, SCPCs with robust AR positive IHC or retained ARS, and samples archived over 10 years in FFPE. We then selected the 4 atypical SCPCs with retained ARS, excluding the outlier 57914. LIMMA produced 1624 differentially expressed genes between prototypical categories, 118 between atypical SCPC and prototypical adenocarcinoma, and 115 between atypical and prototypical SCPC (Additional file 3: Figure S7). Most differentially expressed genes involving atypical SCPC were already differentially expressed between prototypical categories (79/118 and 97/115 genes; p=1.7e-63 and 4.8e-95), with greatest enrichment for “cell cycle phase” (p=1.9e-28) and including known NEPC-related epigenetic genes (EZH2, DNMT1, HIST1H4C). Thus, atypical SCPCs demonstrated a hybrid or intermediate phenotype.

Nearest centroid classification based on the 3 newly defined cohorts and common genes between > 2 pair-wise LIMMA comparisons (Table 6) achieved an estimated error rate of 4.5% (1/22), with incorrect prediction of the atypical SCPC training sample 56107 (although correct classification before LOOCV). On remaining non-training samples, 4/10 classified discordantly with diagnoses: the meta-9 outliers (57914, 57634) and 2/5 mixed adenocarcinomas (56321_A as atypical SCPC, 56104_A as prototypical SCPC; also under models derived after excluding their matched SCPC from training). Behavior of mixed adenocarcinomas, especially considering biopsies, may thus potentially be prognostic of an underlying undetected SCPC component in a subset of cases presumably enriched for mixed tumors with shared clonal driver alterations. On the other hand, 56104_A may have contained an admixed population of SCPC cells as discussed earlier, and if so, it is possible its true adenocarcinoma component might no longer be prognostic.

**Table 6:** Differentially expressed genes common to 2 or more LIMMA comparisons between: 9 prototypical SCPC, 9 prototypical AdCa, and 4 atypical SCPC, with associated centroids

Gene	AD	SC	AS	AD (SCAN)	SC (SCAN)	AS (SCAN)
TPX2	5.40	6.73	7.41	0.16	1.10	1.33
CHEK1	4.42	5.54	6.12	0.02	0.46	0.55
CKAP2L	4.67	5.57	5.70	-0.04	0.41	0.29
HMMR	3.99	4.76	5.17	-0.07	0.25	0.30
CDCA2	4.32	5.05	5.45	-0.13	0.19	0.29
KIF15	4.16	5.15	5.26	-0.07	0.41	0.39
ARHGAP11B	5.11	5.94	6.23	0.05	0.44	0.51
ANLN	4.60	5.57	6.10	0.00	0.49	0.61
WDHD1	4.35	5.07	5.75	0.02	0.31	0.47
NCAPG2	4.60	5.37	5.73	0.03	0.39	0.48
TMPO	5.88	6.74	7.37	0.30	0.97	1.21
KIF2C	4.60	5.21	5.29	-0.11	0.14	0.15
CASC5	4.35	5.18	5.44	-0.04	0.43	0.44
DLGAP5	4.21	5.49	5.96	-0.04	0.68	0.73
TIMELESS	5.28	5.65	6.04	-0.06	0.14	0.28
HMGB2	7.22	8.47	9.12	0.29	0.84	0.94
IQGAP3	5.49	6.18	6.25	-0.04	0.38	0.35
MKI67	5.15	6.51	6.97	0.01	0.90	0.96
STMN1	5.94	7.12	7.23	0.11	0.92	0.91
PBK	3.80	4.85	5.34	-0.12	0.28	0.34
SKA3	4.30	5.49	5.85	-0.02	0.56	0.63
ARHGAP11A	4.51	5.49	5.87	-0.10	0.37	0.36
TOP2A	5.21	6.94	7.84	0.16	1.37	1.66
E2F7	5.17	5.91	6.05	-0.08	0.33	0.30
HJURP	5.65	6.67	6.77	0.00	0.54	0.55
CDC7	4.15	4.97	5.27	-0.07	0.27	0.34
BRIP1	4.03	4.92	5.46	-0.08	0.36	0.52
KIF11	3.94	4.95	5.49	-0.09	0.49	0.58
UBE2C	6.45	7.38	7.59	0.12	0.96	1.12
PTTG1	7.10	9.16	9.20	0.23	1.25	1.08
NCAPG	4.46	5.81	6.25	0.11	0.90	0.95
HIST1H2AJ	7.10	8.15	8.55	0.32	0.99	1.27
CENPF	4.86	6.51	6.96	-0.02	0.90	1.00
ASPM	4.27	5.74	6.28	-0.06	0.71	0.83
CIT	5.29	6.02	6.17	-0.06	0.38	0.38
NEK2	4.90	5.63	5.98	-0.06	0.24	0.33
CDKN2C	5.32	6.21	6.37	-0.19	0.19	0.25
HIST1H2BO	6.51	8.04	7.88	-0.08	1.06	1.32
MELK	4.20	5.26	5.54	-0.04	0.58	0.53
NDC80	4.20	5.54	5.82	-0.02	0.65	0.63
HIST1H4C	7.86	9.06	9.35	1.25	2.35	2.26
CENPK	4.11	5.32	5.94	-0.01	0.47	0.57
GINS1	4.49	5.59	6.22	-0.11	0.43	0.66
CDKN3	4.81	6.06	6.81	-0.03	0.49	0.59
CNIH2	6.08	6.60	6.84	0.04	0.28	0.33
ESCO2	4.06	4.55	4.69	-0.17	0.00	0.05
NUF2	4.54	5.79	5.66	-0.01	0.57	0.35
NUSAP1	5.47	6.91	7.45	0.15	1.15	1.25
KIF23	4.74	5.64	5.85	-0.01	0.46	0.38
CDK1	4.10	5.08	5.22	-0.04	0.40	0.41
FBXO5	5.15	6.19	6.63	0.09	0.56	0.63
CDC20	5.95	6.55	6.71	0.07	0.39	0.45
PLK4	3.94	4.78	5.03	-0.03	0.40	0.39
HIST1H3B	4.87	6.59	7.29	0.19	1.44	1.71
CCNB1	4.96	6.11	6.57	0.05	0.70	0.80
SGOL1	3.99	4.95	5.07	-0.01	0.59	0.54
CENPE	3.83	4.78	5.17	-0.09	0.36	0.42
DEPDC1B	4.43	5.51	5.59	-0.11	0.33	0.27
CLSPN	4.54	5.82	5.69	-0.10	0.53	0.40
CENPW	4.30	5.64	6.14	-0.09	0.50	0.72
FANCI	4.42	5.30	5.67	-0.01	0.46	0.48
LIN9	4.25	4.86	5.26	-0.09	0.14	0.22
DNMT1	6.61	7.25	7.39	0.31	0.71	0.72
KIF4B	5.74	6.30	6.38	0.04	0.48	0.72
ESPL1	5.26	5.77	5.90	-0.14	0.10	0.17
EZH2	5.48	6.38	6.61	0.14	0.68	0.68
LMNB1	5.34	6.66	6.91	0.04	0.63	0.67
CEP55	4.50	5.39	5.40	-0.15	0.25	0.18
WDR76	4.79	6.06	6.25	0.06	0.69	0.69
TUBB2B	7.07	8.17	7.90	-0.08	1.18	0.78
DTL	4.53	5.71	6.23	-0.05	0.56	0.69
KIF18A	3.62	4.73	4.73	-0.16	0.33	0.24
GTSE1	5.29	5.87	5.93	-0.02	0.31	0.31
TROAP	6.58	7.07	7.26	0.12	0.51	0.64
BUB1	4.78	5.78	5.79	-0.06	0.47	0.40
RN7SL720P	3.47	4.19	4.56	-0.35	-0.14	-0.04
SPAG5	5.40	6.11	6.53	0.01	0.39	0.47
MYLK-AS1	6.94	6.22	6.00	0.77	0.41	0.37
CCND1	8.13	7.08	7.32	0.89	0.23	0.44
KLK3	9.85	6.40	9.58	2.87	0.22	2.21
KLK2	9.81	6.22	9.51	2.56	0.12	2.13
ZNF615	6.56	4.71	6.84	0.89	0.06	0.96
TMPRSS2	8.25	5.02	8.01	1.74	-0.01	1.36
NKX3-1	8.29	6.44	8.41	1.19	-0.24	1.25
PMEPA1	7.94	6.31	7.89	1.49	0.29	1.28
HOXB13	8.50	5.43	8.52	1.34	0.08	1.17
KLK4	8.85	6.55	8.85	1.92	0.15	1.70
SNORA59A	5.84	4.94	7.22	0.92	0.35	1.23
ACPP	8.58	4.79	8.04	2.35	0.04	1.59
FOLH1	7.34	4.66	7.25	1.80	0.06	1.37
TRGC1	9.10	4.73	9.17	1.34	-0.02	1.02
ZNF350	6.80	4.77	6.51	0.94	0.11	0.74
BMPR1B	6.51	4.79	7.32	0.99	-0.01	1.28
DSC2	6.29	4.94	6.98	0.57	0.02	0.93
PDE3B	6.41	5.10	6.27	0.60	0.08	0.58
FOLH1B	7.23	4.52	7.07	1.42	-0.05	0.97
ZNF613	7.51	5.95	7.51	1.09	0.44	1.02
RNF138P1	7.21	3.64	7.50	1.71	0.19	1.76
GCNT2	5.33	4.64	5.97	0.22	-0.02	0.44
SLC45A3	8.18	6.42	8.02	1.44	0.04	1.10
PRR16	6.75	5.41	7.02	0.72	0.10	0.81
ZNF614	5.88	4.77	6.04	0.33	0.01	0.47
TULP3	6.15	5.68	6.64	0.67	0.54	0.91
MID2	6.22	5.14	6.20	0.53	0.05	0.51
ZG16B	8.30	7.01	8.42	0.72	0.23	0.72
TRGC2	7.46	4.36	7.37	1.52	-0.03	1.27
ERGIC1	8.17	6.53	8.02	1.37	0.45	1.21
NCOR1	7.25	6.36	7.50	0.92	0.52	1.03
SPDEF	7.90	6.44	7.50	0.96	-0.02	0.66
ALG13	5.81	5.02	6.09	0.66	0.30	0.76
POTEF	7.68	5.37	8.41	1.43	0.36	1.32
GRHL2	7.38	5.24	7.13	1.14	0.14	0.96
SH3RF1	7.21	6.09	7.56	0.93	0.22	1.08
CREB3L4	7.20	5.96	7.23	0.94	0.20	0.90
CAMKK2	7.28	6.14	7.35	0.77	0.20	0.85
WNK1	7.56	6.79	7.91	1.12	0.77	1.38
ZNF649	8.19	6.41	7.90	1.37	0.48	1.19
PPAP2A	7.68	5.97	8.46	1.54	0.43	1.85
C1orf116	7.44	5.93	7.40	1.03	0.20	1.01
ABCC4	7.86	5.32	6.61	1.61	0.07	0.66
ARHGAP6	6.48	5.43	6.79	0.50	-0.11	0.55
RAB27B	5.92	4.77	6.09	0.65	0.09	0.65
ZNF432	6.24	5.02	6.49	0.68	0.18	0.72
ENOSF1	6.27	5.51	6.90	0.48	0.19	0.75
AMELX	5.64	4.66	6.33	0.49	-0.09	0.65
SLC30A4	7.90	5.40	7.18	1.54	0.09	0.91
THRB	5.94	5.27	6.10	0.33	0.04	0.40
HEATR5B	5.58	4.92	5.68	0.48	0.22	0.52
CROT	5.11	4.44	5.11	0.31	0.02	0.23
SLC44A4	8.12	6.08	7.98	1.24	0.14	1.16
TTC19	6.74	6.02	7.26	0.79	0.43	0.87
AADAT	5.86	4.61	6.39	0.62	0.13	0.85
ZNF616	5.48	4.65	5.71	0.37	0.16	0.49
CCDC160	4.51	2.93	4.73	0.26	-0.10	0.27
STEAP2	8.17	5.62	7.86	2.06	0.25	1.58
ADPRM	5.11	4.37	5.25	0.28	-0.01	0.33
TBX3	7.10	6.03	7.56	0.63	-0.04	0.90
AR	7.65	6.02	7.84	1.05	0.16	1.15
EEF2	9.77	8.60	9.66	2.47	1.48	2.22
ZNF880	6.65	5.81	6.93	0.55	0.22	0.76
HERC3	6.69	5.54	6.71	0.97	0.32	0.87
NEDD4L	7.45	5.39	7.06	1.11	0.20	0.78
ARSD-AS1	6.76	5.14	6.72	1.69	0.38	1.60
ZNF33A	5.91	5.06	6.29	0.55	0.33	0.71
MIOS	6.13	4.91	6.48	0.74	0.28	0.84
CPNE4	7.81	4.49	6.25	1.66	0.00	0.72
ARSD	6.71	5.61	6.72	0.14	-0.20	0.14
IQGAP2	5.76	4.62	5.57	0.53	0.03	0.38
ACACA	7.16	5.69	7.38	1.08	0.34	1.15
MALT1	6.63	4.97	6.44	1.11	0.15	0.86
KIAA1551	6.74	5.97	7.04	1.02	0.73	1.35
KIAA1244	7.94	6.21	7.93	1.31	0.31	1.26
REPS2	7.03	5.62	7.20	0.75	0.05	0.71
RASSF3	6.16	5.16	6.40	0.75	0.20	0.88
ADIPOR2	6.74	6.21	7.09	0.53	0.33	0.73
CLK4	6.11	4.96	6.33	0.62	0.25	0.69
AMD1	7.13	5.86	7.68	1.24	0.48	1.45
SLC35F2	5.68	4.57	5.47	0.05	-0.24	-0.04
SCMH1	7.15	6.38	7.33	0.93	0.52	1.00
ALDH6A1	6.38	5.63	6.33	0.58	0.16	0.51
PDE9A	7.15	5.59	6.73	0.73	0.02	0.54
KDM5A	5.97	5.32	6.41	0.44	0.27	0.67
ANKRD50	6.73	5.65	7.62	0.90	0.50	1.52
C1orf21	7.85	6.74	8.10	1.02	0.45	1.16
GNB2L1	10.03	8.98	10.40	1.33	1.00	1.49
RANBP3L	5.85	4.26	6.10	0.71	0.00	0.76
IGBP1	6.68	6.16	6.97	0.63	0.40	0.79
PPAPDC1B	7.17	6.12	7.02	0.78	0.11	0.63
MSMB	8.43	4.45	8.34	2.39	0.03	1.94
C12orf4	5.08	4.50	5.45	0.33	0.09	0.50
ZNF577	7.30	5.57	6.78	1.16	0.28	0.90
ZNF841	6.67	5.55	6.69	1.15	0.51	1.11
RN7SL97P	3.75	4.77	3.57	-0.45	-0.16	-0.54
POTEH	8.09	4.74	9.36			
POTEH-AS1	7.54	5.60	7.79			
FAM115A	6.88	6.04	6.98			

Legend: Cohorts of 9 prototypical adenocarcinomas, 9 prototypical SCPCs, and 4 atypical SCPCs were formed from meta-9 clusters after removing outliers, mixed adenocarcinomas, adenocarcinomas associated with NE differentiation, and samples archived over 10 years in FFPE. Differentially expressed genes were calculated by LIMMA for all possible pair-wise comparisons of cohorts, and filtered for genes shared by 2 or more comparisons. This resulted in 176 genes consisting of 79 genes (77 up, 2 down) differentially expressed in common between either prototypical SCPCs or atypical SCPCs versus prototypical adenocarcinomas, and 97 genes (1 up, 96 down) differentially expressed in common between prototypical SCPCs and either prototypical adenocarcinomas or atypical SCPCs. Centroids for each cohort (values in the table) were formed for this gene-set and transferred to the GRID database under SCAN normalization for use in a nearest-centroid classifier model

**Fig. 6:**
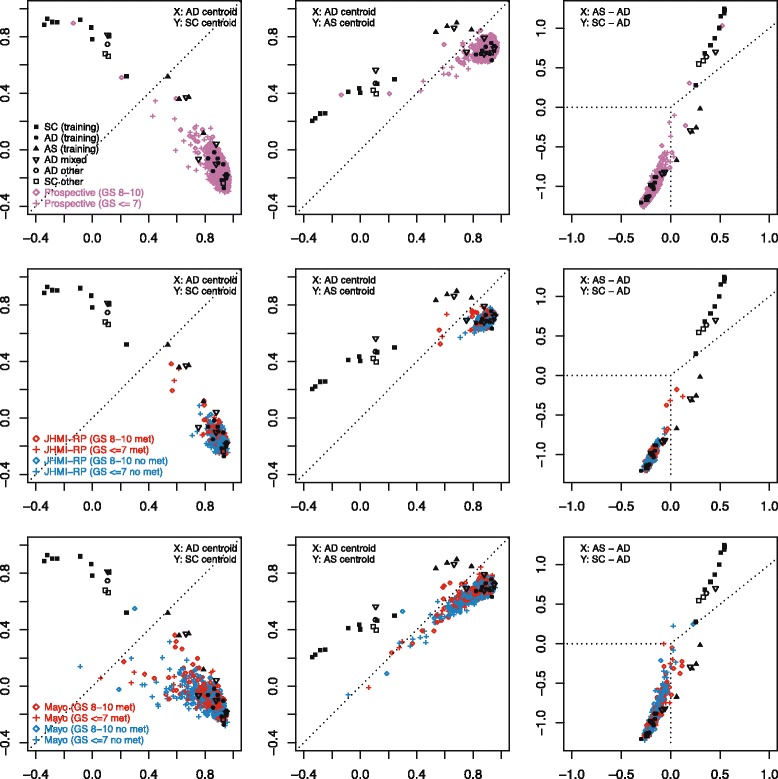
Nearest 3-centroid classification of GRID® RP adenocarcinoma cohorts. We assessed performance of nearest centroid classification in GRID® RP adenocarcinoma cohorts Prospective (N=2993), JHU-RP (N=355), and Mayo (N=780) relative to centroids AD (prototypical adenocarcinoma), SC (prototypical SCPC), and AS (atypical SCPC). Two Prospective (0.09%) and no JHU-RP (0%) samples were classified as prototypical SCPC while 4 Prospective (0.17%) and 2 JHU-RP (0.6%) samples were classified as atypical SCPC. A greater proportion of Mayo samples (1.6%) were classified as SCPC but likely included false positives with low correlations to all 3 centroids

We transferred the 3-centroid classifier to the GenomeDx GRID**®** by reformulating centroids under SCAN (Table 6), a single-sample normalization method compatible with routine clinical lab environments although susceptible to batch effects. JHU-FFPE samples were handled relatively uniformly, yet demonstrated notable effects based on RNA processing date (Additional file 3: Figure S8); nevertheless SCAN (compared to RMA) empirically produced identical classification of JHU-FFPE, suggesting robustness. We applied the 3-centroid model to selected GRID**®** adenocarcinoma cohorts, and found that 2 Prospective (0.09%), no JHU-RP (0%), and 2 Mayo (0.3%) samples classified as prototypical SCPC, and 4 Prospective (0.17%), 2 JHU-RP (0.6%), and 10 Mayo (1.3%) samples classified as atypical SCPC (Fig. 6). Both JHU-RP samples with atypical SCPC classification were part of a distinct cluster of 4 samples featuring the highest CCP and 3 lowest ARS scores among JHU-RP, and all 4 subsequently developed metastases. Mayo had greater proportions classifying as SCPC but included suspected false positives far from training samples with low correlations to all 3 centroids. In the earlier meta-12 analysis (Fig. 2), the Mayo-FFPE dataset similarly exhibited multiple samples with low correlations to both meta-12 centroids. Mayo samples overall also had weaker correlations to the adenocarcinoma centroid (r=0.82) versus samples from Prospective (r=0.92) or JHU-RP (r=0.89).

We remark that our JHU-FFPE datasets had variable archive ages (Table 3), which potentially impacted expression and is discussed further in the next section. SCPCs and adenocarcinomas were at least relatively balanced (mean 3.6 and 3.0 years after removing the oldest sample), ideally minimizing differential bias. By contrast, cohorts demonstrated a few notable differences in tissue sources, for example pure adenocarcinomas were all biopsies. This potentially affected both expression and differential expression, however we at least found no significant differences by LIMMA between biopsies and TURPs (the most common sources) when restricted to SCPCs, or among all samples.

### FFPE introduces an extra source of variability to the JHU-FFPE dataset

**Fig. 7:**
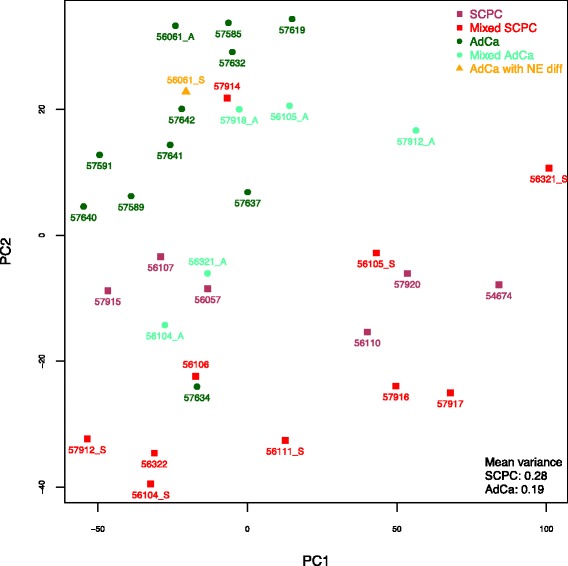
Principal components analysis of JHU-FFPE. SCPCs and adenocarcinomas were generally separated by principal components analysis. Mixed adenocarcinomas exhibited roughly intermediate behavior, although we questioned whether 56104_A contained an accidental admixture with its neighboring small cell component 56104_S. One SCPC (57915) and one pure adenocarcinoma (57634) clustered with opposite phenotypes, similar to meta-9 clustering. Among SCPCs with known AR or PSA-positivity, two clustered side by side in a relatively intermediate territory (57915, 56107) while the third was loosely in the vicinity (57912_S). Of all principal components, PC2 separated SCPCs from adenocarcinomas best (AUC 86%) and was highly correlated to the difference between ARS and CCP (r=0.93). By contrast in frozen tissue primary and xenograft NEPC datasets, respective PC1's separated SCPCs from adenocarcinomas best (AUC 100%) and was highly correlated to the difference between CCP and ARS (r=0.75-0.98) (Supp Figure 9). Thus in JHU-FFPE, PC1 represented a different source of greatest variability. Examination of its top coefficients by magnitude revealed that PC1 was highly anti-correlated to the average expression of various ribosomal subunits (r = -0.93) including RPL19, known to be an effective reference gene. Two SCPCs (56321_S, autopsy 54674) had the largest PC1 magnitudes and were archived 14-16 years (versus 0-6 years for other SCPCs), perhaps reflecting higher levels of RNA degradation; however the oldest adenocarcinoma (56321_A) did not exhibit this trend

Principal components analysis of JHU-FFPE, considering all genes for an unsupervised approach, demonstrated rough separation of phenotypes, intermediate behavior of mixed adenocarcinomas, and discordant behavior of the meta-9 outlier samples (57914, 57634) (Fig. 7). Of all 33 principal components, the second (PC2) best separated phenotypes (AUC 86.3%) and had the greatest magnitude correlations to each of CCP (r = -0.88), ARS (r=0.69), and neuronal phenotype scores (r=-0.54), with higher correlation to the difference of ARS and CCP (r=0.93). Indeed, under GSEAPreranked applied to the PC2 gene coefficients, NELSON-RESPONSE-TO-ANDROGEN-UP was the #2 most up-regulated gene-set (out of 3739 gene-sets from the Molecular Signatures Database curated collection C2 after size filters), while the top down-regulated gene-sets were largely cell cycle related (ROSTY-CERVICAL-PROLIFERATION-CLUSTER was #1, REACTOME-CELL-CYCLE was the top Reactome pathway at #28, and KEGG-CELL-CYCLE was the top KEGG pathway at #82). By contrast, in principal component analyses of the 4 frozen tissue primary or xenograft NEPC datasets, the first principal component (PC1) always separated NEPCs from adenocarcinomas (AUC 100%) (Additional file 3: Figure S9), and moreover always had the greatest magnitude correlations to ARS (r=-0.76 to -0.98), neuronal phenotype (r=0.87 to 0.98), and CCP scores (r=0.57 to 0.93), with the exception of CCP in 1/4 datasets.

Thus in JHU-FFPE, its first principal component (PC1, representing the direction of greatest variability) appeared to include a different source of variability. While still demonstrating moderate correlations to ARS (r=-0.62) and neuronal phenotype (r=0.46) and to lesser degree to CCP (r=-0.20), PC1 did not separate phenotypes very well (AUC 61.7%), and its greatest magnitudes were notably from SCPCs of oldest FFPE age (54674, 56321_S), both archived 14-16 years (versus 0-6 years for other SCPCs). There was moderate correlation between PC1 and archive age (r=0.50), and PC1 modestly differentiated older archived samples (> 3y in FFPE) versus newer samples (p=0.04). We also tested whether PC1 was associated with sample type (biopsies versus TURPs) but did not find evidence for this (p=0.48). We applied GSEAPreranked to better characterize the source of variability captured by PC1. The most down-regulated gene-sets were related to RNA translation (REACTOME-SRP-DEPENDENT-COTRANSLATIONAL-TARGETING-TO-MEMBRANE was #1 while KEGG-RIBOSOME was the top KEGG pathway at #5). Eighteen of the top 100 gene coefficients by magnitude corresponded to ribosomal protein subunits, with PC1 highly anti-correlated to their average gene expression (r=-0.93). These genes included RPL19, which has been used previously in FFPE gene expression analysis to normalize sample input [40]. Up-regulated gene-sets were considerably rarer (47 versus 2104 with nominal p-val < 0.01), and included epigenetic-related gene-sets (e.g., KONDO-PROSTATE-CANCER-WITH-H3K27ME3 was #3).

Given the possible influence of the variable archive ages in JHU-FFPE on gene expression, we attempted to investigate individual gene performance. Since the exon array contained probe-sets for almost every exon of a gene, probe-sets targeting the same transcript ideally behaved concordantly, and we defined correlation strength (CS) as average correlation between probe-sets targeting the same gene and restricted here to genes with 10 or more probe-sets. CS was considerably weaker in FFPE datasets versus a frozen tissue dataset, with decline related to archive age and presumably to RNA degradation (Additional file 3: Figure S10). In JHU-FFPE, CS was lower for neuronal phenotype genes (mean 0.24) versus cell cycle progression genes (0.36) or AR-signaling genes (0.56), consistent with the relative paucity of neuronal genes in differential expression analysis. For instance, CHGA had relatively weak CS versus frozen tissue (CS=0.31 versus 0.77), while androgen-related genes (KLK3, KLK2, ACPP) had the highest CS (0.86-0.88) and standard deviations (1.90-1.95) (Additional file 3: Figure S11). Accuracy in FFPE has been reported to improve upon using each gene’s most variable probe-set [16]. Compatible with this, CS increased on average by 0.14 in JHU-FFPE upon restricting to each gene’s 5 most variable probe-sets, likely through exclusion of weakly binding, oversaturated, or unused alternative exon probe-sets. We also investigated expression in JHU-FFPE of the gene CCEPR, elevated in 88% of NEPCs in the meta-analysis. CS no longer applied since only one exon probe-set (3290641) targeted CCEPR. This probe-set did not differentiate between phenotypes (nominal p=0.54 compared with its neighbor PHYHIPL p=0.05), had relatively narrow dynamic range, and lost correlation to PHYHIPL (r=0.17 versus 0.71 in NIH Roadmap data), suggesting poor performance in FFPE.

## Discussion

We utilized an outlier-based meta-analysis approach to study prototypical high-grade NEPC across multiple frozen tissue datasets, although more sophisticated methods have also been described [41]. We believe meta-12 centroids may provide a useful tool to assess for prototypical high-grade NEPC status given high quality frozen tissue expression data, however we also found evidence of highly similar meta-12 centroid correlation profiles between prototypical high-grade NEPC and small cell carcinomas from lung and possibly other sites, reflecting the challenge of determining site of origin in small cell carcinoma of unknown primary. Although we did not validate individual genes in this study, e.g. via PCR or RNA in-situ hybridization, we believe meta-12 genes are strong candidates for potential diagnostic markers, either through RNA or protein; in a previous study, we found that cyclin D1 performed effectively as a negative IHC marker of SCPC [21], and further evaluation of selected meta-12 genes, both up and down, is currently underway.

We provided one of the largest gene expression datasets to date of primary SCPC and high-grade adenocarcinoma, albeit in FFPE, including significant proportions of mixed SCPCs (63%), slightly above estimates from the literature (40-50%), and SCPCs with preserved AR signaling (31%), slightly above reported frequencies of AR-positive or PSA-positive SCPC (17-20%) [5, 6]. Based on meta-signature-derived subgroups of this dataset, we developed a nearest 3-centroid classifier for primary samples profiled by exon array. One adenocarcinoma, with highly aggressive metastatic progression described in a previous case report, was classified as prototypical SCPC. Two mixed adenocarcinomas (40%) were additionally classified as SCPC (1 prototypical, 1 atypical), suggesting that mixed cases might be enriched for SCPC signatures in their adenocarcinoma components, due perhaps to shared clonal origins although possibly false positives from admixture. The classifier may thus provide utility for detection of mixed cases in the biopsy setting, where only the adenocarcinoma component might get sampled.

Rare adenocarcinomas among GRID**®** cohorts were also classified as SCPCs under the 3-centroid model, similar to behavior of the JHU-FFPE outlier or unusual mixed adenocarcinomas. Percentages of such GRID**®** cases (0.3-0.6%, excluding Mayo due to suspected false positives) were generally below the presumptive frequency of SCPC (often reported as 0.5-2%) [42], roughly in line with expectations given that GRID**®** cohorts consisted of RP adenocarcinomas and inherently excluded SCPCs. We suspect these cases may correspond to diagnostically challenging poorly differentiated tumors, misdiagnosed samples, mixed adenocarcinomas, or fortuitously sampled occult SCPC components, however further investigation is necessary. Cases were also too scarce for meaningful Kaplan-Meier analysis, however the 2 JHU-RP cases with atypical SCPC classification belonged to a cluster of 4 cases that all subsequently developed metastases. Thus, we speculate the classifier may detect unusually aggressive cases and potentially have prognostic relevance.

One main limitation of our study was the lack of an independent validation set of primary SCPCs to test the 3-centroid classifier. In contrast to the multiple large GRID**®** adenocarcinoma cohorts, few SCPCs have been profiled on the GRID**®**, due to rarity of diagnosis and also scarcity of tissue, given that SCPC patients have traditionally been treated with systemic therapy (usually after biopsy-based diagnosis) and not with RP. Moreover, patients found to have unexpected SCPC upon RP would typically have little need for prognostic clinical RNA expression testing on the GRID**®**. Consequently we were not aware of other exon array datasets with annotated SCPCs. However, it was at least encouraging that the 2 JHU-FFPE SCPCs excluded from training due to old archive age were indeed classified as prototypical SCPCs despite their outlier PCA trends.

Another limitation of the classifier was its derivation from relatively few atypical SCPCs, indicating a need for more samples to definitively establish whether cases such as 57912_S with a uniform hybrid IHC pattern and small cell morphology are indeed a true subcategory with common underlying genomic properties. Similarly, the pattern of low ARS without neuronal over-expression may deserve a separate category in the primary or metastatic setting, but also requires more examples. Such non-standard cases, often manifesting as hybrid or unusual IHC profiles, can be puzzling for pathologists to evaluate. The ultimate clinical question will be whether these potential expression-based subtypes have prognostic relevance or predict response to therapy. Anecdotally, the outlier adenocarcinoma in our JHU-FFPE dataset with low ARS and non-elevated neuronal expression had unusually aggressive metastatic progression described in a case report [32]. We did not have access to outcome data of the atypical hybrid SCPCs in our dataset and were not aware of other hybrid SCPCs in the literature, however rare adenocarcinoma cases with aggressive progression and hybrid IHC co-expression of AR and chromogranin have been reported [9, 10]. There is also increasing evidence for lineage plasticity between adenocarcinoma and neuroendocrine phenotypes in metastatic prostate cancer, induced upon anti-androgen therapy and partially reversed through epigenetic interventions such as EZH2 inhibition [43–45]. Our atypical hybrid SCPCs, as well as the outlier adenocarcinoma, overexpressed epigenetic genes including EZH2. We hope increased recognition of these unusual phenotypes will lead to larger collections of cases and eventual clarity on their clinical relevance.

## Conclusions

Meta-analysis generates a robust signature of prototypical high-grade NEPC, with close resemblance to small cell lung cancer. Atypical NEPC potentially includes a hybrid subcategory exhibiting preserved AR-signaling and a non-neuronal subcategory with AR loss and high proliferation but without expression of neuroendocrine markers that may overlap with adenocarcinomas. In the primary setting, FFPE material may be used to generate a classifier of SCPC incorporating disease heterogeneity, with potential prognostic implications. However, further testing with a proper validation set is required.

## Additional Files

Additional file 1: Additional methods on bioinformatic processing and analysis, and additional legends. (DOCX 49 kb)

Additional file 2: Additional tables on NEPC gene-lists, meta-9 genes, and LIMMA comparisons. (XLSX 266 kb)

Additional file 3: Additional figures on meta-12 scores, AR signaling versus AR / CCP / RAB3B, mixed tumors, REST exons, batch effects, principal components, and correlation strengths. (PDF 13484 kb)

## Abbreviations

AdCa (or Ad): Adenocarcinoma
ARS: AR-signaling (gene signature)
CCP: Cell cycle progression (gene signature)
CRPC: Castration-resistant prostate cancer
FFPE: Formalin-fixed paraffin-embedded
LCNEC: Large cell neuroendocrine carcinoma
LOOCV: Leave-one-out cross-validation
NED: Neuroendocrine differentiation
NEPC: Neuroendocrine prostate cancer
SCLC: Small cell lung cancer
SCPC: Small cell prostatic cancer

## References

[1] Epstein, JI.,et al., Proposed morphologic classification of prostate cancer with neuroendocrine differentiation. Am J Surg Pathol, 2014. 38(6): p. 756-767. 10.1097/PAS.0000000000000208 PMC4112087 24705311

[2] Wang HT (2014). Neuroendocrine prostate cancer (nepc) progressing from conventional prostatic adenocarcinoma: factors associated with time to development of nepc and survival from nepc diagnosis-a systematic review and pooled analysis. J Clin Oncol.

[3] Tan, HL, et al., Rb loss is characteristic of prostatic small cell neuroendocrine carcinoma. Clin Cancer Res, 2014. 20(4): p. 890-903. 10.1158/1078-0432.CCR-13-1982 PMC3931005 24323898

[4] Lapuk AV (2012). From sequence to molecular pathology, and a mechanism driving the neuroendocrine phenotype in prostate cancer. J Pathol.

[5] Yao JL (2006). Small cell carcinoma of the prostate: an immunohistochemical study. Am J surg pathol.

[6] Wang W, Epstein JI. Small cell carcinoma of the prostate. A morphologic and immunohistochemical study of 95 cases. Am J Surg Pathol. 2008;32(1):65–71. 10.1097/PAS.0b013e318058a96b 18162772

[7] Kumar A (2016). Substantial interindividual and limited intraindividual genomic diversity among tumors from men with metastatic prostate cancer. Nat Med.

[8] Beltran H (2016). Divergent clonal evolution of castration-resistant neuroendocrine prostate cancer. Nat Med.

[9] Roudier MP (2004). Metastatic conventional prostatic adenocarcinoma with diffuse chromogranin a and androgen receptor positivity. J Clin Pathol.

[10] Wu C (2012). Integrated genome and transcriptome sequencing identifies a novel form of hybrid and aggressive prostate cancer. J Pathol.

[11] Beltran H (2011). Molecular characterization of neuroendocrine prostate cancer and identification of new drug targets. Cancer Discov.

[12] Lin D (2014). High fidelity patient-derived xenografts for accelerating prostate cancer discovery and drug development. Cancer Res.

[13] Tzelepi V (2012). Modeling a lethal prostate cancer variant with small-cell carcinoma features. Clin Cancer Res.

[14] Zhang X (2015). Srrm4 expression and the loss of rest activity may promote the emergence of the neuroendocrine phenotype in castration-resistant prostate cancer. Clin Cancer Res.

[15] Hansel DE (2009). Shared tp53 gene mutation in morphologically and phenotypically distinct concurrent primary small cell neuroendocrine carcinoma and adenocarcinoma of the prostate. Prostate.

[16] Gravendeel LA (2012). Gene expression profiles of gliomas in formalin-fixed paraffin-embedded material. Br J Cancer.

[17] Abdueva D (2010). Quantitative expression profiling in formalin-fixed paraffin-embedded samples by affymetrix microarrays. J Mol Diagn.

[18] Hieronymus H (2006). Gene expression signature-based chemical genomic prediction identifies a novel class of hsp90 pathway modulators. Cancer cell.

[19] Cuzick J (2011). Prognostic value of an rna expression signature derived from cell cycle proliferation genes in patients with prostate cancer: a retrospective study. Lancet Oncol.

[20] Ritchie ME (2015). Limma powers differential expression analyses for rna-sequencing and microarray studies. Nucleic Acids Res.

[21] Tsai H (2015). Cyclin d1 loss distinguishes prostatic small-cell carcinoma from most prostatic adenocarcinomas. Clin Cancer Res.

[22] Travis WD. Update on small cell carcinoma and its differentiation from squamous cell carcinoma and other non-small cell carcinomas. Mod Pathol. 2012;(25 suppl 1):S18–30. 10.1038/modpathol.2011.150 22214967

[23] Dalela D (2016). Contemporary role of the decipher(r) test in prostate cancer management: current practice and future perspectives. Rev Urol.

[24] Karnes RJ (2013). Validation of a genomic classifier that predicts metastasis following radical prostatectomy in an at risk patient population. J Urol.

[25] Erho N (2013). Discovery and validation of a prostate cancer genomic classifier that predicts early metastasis following radical prostatectomy. Plos One.

[26] Ross AE (2016). Tissue-based genomics augments post-prostatectomy risk stratification in a natural history cohort of intermediate- and high-risk men. Eur Urol.

[27] Glass AG (2016). Validation of a genomic classifier for predicting post-prostatectomy recurrence in a community based health care setting. J Urol.

[28] Grasso CS (2012). The mutational landscape of lethal castration-resistant prostate cancer. Nature.

[29] Sircar K (2012). Mitosis phase enrichment with identification of mitotic centromere-associated kinesin as a therapeutic target in castration-resistant prostate cancer. Plos One.

[30] Yang M (2015). Long noncoding rna cche1 promotes cervical cancer cell proliferation via upregulating pcna. Tumour Biol.

[31] Adler P (2009). Mining for coexpression across hundreds of datasets using novel rank aggregation and visualization methods. Genome biol.

[32] Haffner MC (2015). Diagnostic challenges of clonal heterogeneity in prostate cancer. J Clin Oncol.

[33] Tan PY (2012). Integration of regulatory networks by nkx3-1 promotes androgen-dependent prostate cancer survival. Mol cell biol.

[34] Rajan P (2014). Next-generation sequencing of advanced prostate cancer treated with androgen-deprivation therapy. Eur urol.

[35] Sharma A (2010). The retinoblastoma tumor suppressor controls androgen signaling and human prostate cancer progression. J clin invest.

[36] Akamatsu S (2015). The placental gene peg10 promotes progression of neuroendocrine prostate cancer. Cell rep.

[37] Palm K, Metsis M, Timmusk T. Neuron-specific splicing of zinc finger transcription factor rest/nrsf/xbr is frequent in neuroblastomas and conserved in human, mouse and rat. Brain res mol brain res. 1999;72(1):30–9. 10.1016/s0169-328x(99)00196-5 10521596

[38] Shimojo M (2013). The small cell lung cancer-specific isoform of re1-silencing transcription factor (rest) is regulated by neural-specific ser/arg repeat-related protein of 100 kda (nsr100). Mol Cancer Res.

[39] Greytak SR (2015). Accuracy of molecular data generated with ffpe biospecimens: lessons from the literature. Cancer Res.

[40] Yang W (2006). Direct quantification of gene expression in homogenates of formalin-fixed, paraffin-embedded tissues. Biotechniques.

[41] Hughey JJ, Butte AJ. Robust meta-analysis of gene expression using the elastic net. Nucleic acids res. 2015;43(12):E79. 10.1093/nar/gkv229 PMC4499117 25829177

[42] Helpap B, Kollermann J, Oehler U. Neuroendocrine differentiation in prostatic carcinomas: histogenesis, biology, clinical relevance, and future therapeutical perspectives. Urol int. 1999;62(3):133–8. 10.1159/000030376 10529661

[43] Kleb B (2016). Differentially methylated genes and androgen receptor re-expression in small cell prostate carcinomas. Epigenetics.

[44] Ku SY (2017). Rb1 and trp53 cooperate to suppress prostate cancer lineage plasticity, metastasis, and antiandrogen resistance. Science.

[45] Mu P (2017). Sox2 promotes lineage plasticity and antiandrogen resistance in tp53- and rb1-deficient prostate cancer. Science.

