# Supplementary material for: Gene expression signatures of neuroendocrine prostate cancer and primary small cell prostatic carcinoma
Source: BMC Cancer. 2017 Nov 13;17:759. doi: 10.1186/s12885-017-3729-z (PMC5683385; doi:10.1186/s12885-017-3729-z)
Supplement: Supplementary file 1 — Additional methods on bioinformatic processing and analysis, and additional legends. (DOCX 49 kb) [file 12885_2017_3729_MOESM1_ESM.docx]

LEGENDS (Supplementary Figures)

Supp Figure 1: Percentages of meta-12 up and down genes with outlier status across samples of the meta-analysis datasets. (A) Among NEPC samples (SCPC / LCNEC), those from UW-CRPC had the least concordance with respect to the meta-12 genes. This dataset had the largest average variance among its adenocarcinomas, making outlier z-score criteria more difficult to satisfy. (B) The top outlier adenocarcinoma samples (based on proportion of outlier meta-12 genes) were UM-CRPC WA46 (39%) reported to have morphologic features of NE differentiation, UW-CRPC 05-187E1_LIVER (26%) predominantly under-expressing meta-12 down genes, and UW-CRPC 07-042H2_LN (25%) classified as adenocarcinoma with NE differentiation and predominantly over-expressing meta-12 up genes. Other notable outliers included UM-CRPC WA25 (20%) reported to have NE differentiation and squamous morphology, MDA-xenograft 117-9 (19%) classified as adenocarcinoma with NE differentiation, VPC-xenograft LTL-331 (16%) which underwent NEPC transdifferentiation under ADT, and LuCaP-xenograft 86 (12%) known to have homozygous RB loss. These samples also remained among the top outliers with respect to the meta-9 gene-set but with reduced proportions of concordance (data not shown), in line with the expectation that meta-9 genes contained additional but less consistent markers of NEPC.

Supp Figure 2: Meta-12 scores based on nearest centroid (difference of correlations to NEPC and adenocarcinoma centroids), correlation to NEPC centroid, correlation to adenocarcinoma centroid (not shown), and average signed expression. In discovery datasets but not non-discovery datasets, NEPC and adenocarcinoma samples were completely separated (AUC 100%) by suitable cutoffs (centroid correlation difference -0.2, NEPC centroid correlation 0.4, adenocarcinoma centroid correlation 0.6, signed average 0). PLAGE was essentially equivalent to signed average (r>0.99 across datasets) but without uniform cutoff (not shown). Since correlations were attenuated in WCMC and JHU-FFPE, difference of correlations used in nearest centroid scoring possibly partially compensated one another, leading to a more robust cutoff compared to use of a single centroid. In SU2C, misclassified samples based on nearest centroid were identical to those under integrated NEPC classification.

Supp Figure 3: ARS score versus AR gene expression in NEPC datasets. ARS scores were retained at levels similar to adenocarcinomas (fold-change > -0.5 and z-score > -1 relative to adenocarcinomas) in 5/16 (31%) SCPCs from JHU-FFPE, 1/19 (5%) SCPCs from UW mCRPC and UW extra, 1/15 (7%) NEPCs from WCMC mCRPC, 1/5 (20%) NEPCs from SU2C mCRPC, and no (0%) other annotated SCPCs or LCNEC from the remaining datasets. In JHU-FFPE, ARS was retained in both pure and mixed SCPCs (3/6 versus 2/10; p=0.30), which included the SCPCs with robust AR positivity by IHC and unusual hybrid profiles. In UW datasets, one primary SCPC from UW-extra (03-192N) had retained ARS while metastatic samples from the same patient were AR-negative by IHC with reduced ARS [[1](#_ENREF_1)]. In WCMC mCRPC, ARS was retained in one metastatic NEPC (7520_1_N), which was annotated in a previous study as mixed histology (SCPC), PSA negative, and TMPRSS2-ERG positive; this sample was removed before the final derivation of an integrated NEPC gene signature [[2](#_ENREF_2), [3](#_ENREF_3)]. In SU2C, ARS was retained in one NEPC and borderline in another, however both possibly corresponded to adenocarcinomas with NE differentiation.

Supp Figure 4: RAB3B versus AR signaling with color-axis for neuronal phenotype across datasets. Twenty samples with low ARS and low/regular neuronal phenotype were identified based on outlier cut-offs relative to adenocarcinomas (fold-change < -1, z-score < -2 for ARS; fold-change < 0.5, z-score < 1 for neuronal phenotype). This included samples with low ARS from pure adenocarcinoma datasets (MSKCC). RAB3B was the top-most differentially expressed gene in common from comparisons versus remaining adenocarcinomas or versus remaining NEPC samples. RAB3B correlated with androgen signaling among adenocarcinomas but also had increased expression in association with NEPC and the neuronal phenotype.

Supp Figure 5: CCP versus AR signaling in matched RNA-seq data (GSE48403) of prostate biopsies before and after ~22 weeks of ADT from 7 patients with locally advanced or metastatic prostate cancer [[4](#_ENREF_4)]. In every patient, both ARS and CCP decreased after ADT compared to matched RNA-seq data from the prior biopsy. Thus, gene expression patterns of low ARS and low CCP potentially may represent response to therapy.

Supp Figure 6: Tissue microarray (TMA) photographs of a mixed histology tumor with SCPC component 56104_S and adenocarcinoma component 56104_A. Hematoxylin and eosin stains showed unusual trabeculation in 56104_S with morphology consistent with small cell carcinoma versus glandular formation in 56104_A diagnosed as Gleason grade 3+4 adenocarcinoma. IHC studies were generally consistent with diagnoses: positive neuroendocrine markers (chromogranin, synaptophysin, CD56), negative or weak androgen-related markers (PSA, NKX3.1, AR), and negative Rb1 and cyclin D1 for 56104_S, versus opposite findings for 56104_A (Table 3). There were TMA cores containing accidental admixtures of 56104_S and 56104_A, such as the core shown here of 56104_S with AR IHC where glands of 56104_A appeared. This potentially caused contamination in gene expression profiling.

Supp Figure 7: Probe-set level expression in the JHU-FFPE dataset for 26 probe-sets (13 core, 12 extended, 1 full) mapping to the REST locus (introns or exons) with SCPC samples color-coded red or maroon and adenocarcinomas in green. Probe-sets targeting exons towards the 3’ end had noticeably reduced expression in SCPC, and on the gene level REST was differentially expressed under LIMMA (adj p=0.048). Truncated alternative splice variants incorporating the 50-62bp cryptic exon located at chr4:56,927,615-56,927,664 and targeted by the probe-set 2728423 have also been associated with reduced REST activity in small cell lung cancer and neuroblastoma. However, expression levels of probe-set 2728423 were consistently low across samples (mean 2.3, max 4.2) and less than the majority (7 of 8) of intronic probe-sets (means 6.0, 4.6, 2.2, 3.7, 4.1, 2.5, 2.6, 3.6; max 6.9, 5.9, 4.1, 5.6, 5.4, 4.3, 4.9, 4.6), making it difficult to distinguish between truly absent or minimal exon expression versus poor probe-set performance (e.g., due to relatively low GC content 34%). Other tissue datasets using the exon array including NIH Roadmap, ENCODE cell lines, and neuroblastoma SHY5Y (GSE21305) had similarly low levels of the probe-set, and although some samples from fetal brain, retinoblastoma, and neuroblastoma exhibited the highest ratios relative to overall REST, these ratios were still less than levels suggested by RNA-seq data (SRP055027, ERP010791), where SHSY5Y and NEPC xenograft LTL331 showed evidence of alternative splicing in roughly 1:1 ratio to regular splicing (data not shown).

Supp Figure 8: Principal components analysis of JHU-FFPE under SCAN normalization color-coded by RNA processing date. January and June samples were separated by the second principal component (PC2), with over 6000 genes differentially expressed under LIMMA. However, phenotype distribution was uneven, with relative lack of non-mixed adenocarcinomas in the January group. The 2 oldest SCPCs were outliers with the largest magnitudes of PC1, but this was also seen in the batch-corrected RMA-processed data.

Supp Figure 9: Principal components analysis of xenograft and localized NEPC frozen tissue datasets. The first principal component PC1 always demonstrated clear separation of NEPCs from adenocarcinomas (AUC 100%). Out of all principal components in each dataset, PC1 also always had the greatest correlations in absolute magnitude to ARS (r=-0.76 to -0.98), neuronal phenotype (r=0.87 to 0.98), and CCP scores (r=0.57 to 0.93), with the exception of CCP in MDA LOCAL-CRPC (where it had the second highest). NEPC samples had greater average variance per gene than adenocarcinomas in 3 / 4 datasets, and also in JHU-FFPE (Fig 7), however most datasets included few NEPC samples. This trend was true for clinical datasets containing primary tumors (MDA LOCAL and JHU-FFPE) and persisted in JHU-FFPE after exclusion of outlier NUSE samples.

Supp Figure 10: Correlation strengths (CS) across FFPE and frozen tissue datasets. (A) CS was overall weaker in FFPE expression data versus frozen tissue data (p=0), weakest in older Mayo-FFPE data, and increased upon restriction to each gene’s 5 most variable probesets. (B) CS of JHU-FFPE versus MSKCC-metastases, with selected NEPC-related genes (black), showed significant skew (4% versus 43% of genes had CS>0.5). Genes with CS > 0.8 in both datasets (red) were often prostate-specific. Among NEPC-related signatures, CS was highest for ARS genes (mean 0.56), followed by meta-12 down (0.48), CCP (0.36), meta-12 up (0.27) and neuronal genes (0.24) (see Table 4 for meta-12 genes). Ordered CS from JHU-FFPE was also most enriched for androgen response gene-sets under GSEAPreranked.

Supp Figure 11: Probe-set level expression for KLK3 and CHGA with IHC available for 11/33 JHU-FFPE samples scored as positive (+), negative (-), or weak (w). Probe-sets were highly concordant for KLK3 and generally agreed with IHC of its protein product PSA, however unusual SCPCs with robustly positive AR IHC (56107, 57912_S) were negative for PSA but still appeared to express KLK3. KLK3 had one of the highest CS and standard deviations among all genes. By contrast, many probe-sets for CHGA had narrow dynamic range and appeared uninformative (CS=0.31). In comparison, MSKCC had one sample with relatively high expression across almost all CHGA probe-sets (CS=0.77). Restricting to the 5 most variable probe-sets for CHGA in JHU-FFPE increased CS from 0.31 to 0.69.

LEGENDS (Supplementary Tables)

Supp Table 1: Common genes among the 8 literature NEPC gene-lists. To compare gene-sets and identify common genes, we updated gene names and probe assignments with current HGNC symbols, which produced further instances of known NEPC-related genes such as SRRM4 and REST. Where possible, we resolved un-annotated probes and non-standard transcripts through BLAT alignment of underlying sequences to hg19, which revealed an occurrence of the gene CCEPR (in MDA xeno). No genes were common to all lists, with the most frequent comprised of 9 largely neuronal up-genes in 5/8 lists (BSN, CRMP1, GPRIN1, INA, MAST1, MYT1, RAB3C, SNAP25, UNC13A) and 5 largely androgen-related down-genes in 4/8 lists (CYP1B1, KLK2, KLK3, STEAP1, TRPV6).

Supp Table 2: Pair-wise overlaps of gene-sets were evaluated by Fisher exact test as a rough statistical measure of similarity, using a presumed background of ~20000 genes. Nineteen of 28 gene-set pairs had p-value < 1e-4. The recent WCMC mCRPC study, which had the most NEPC patients and generated the largest gene-set, was part of the top 4 most significant pairs, partially reflecting its statistical power. VPC studies formed the other half of the top 2 pairs, with the very top pair based on a xenograft model of neuroendocrine trans-differentiation under ADT, suggesting its similarity to clinical CRPC; however, its gene-list was also based on WCMC 2011 and not completely independent from WCMC mCRPC. The 3rd most significant pair consisted of both WCMC studies sharing 2 NEPC patients. The 4th most significant pair consisted of clinical CRPC studies with primarily metastatic samples and whose NEPC cohorts included adenocarcinomas with NE differentiation. The 5th most significant pair consisted of the 2 studies comparing predominantly metastatic NEPC versus primary adenocarcinoma. By contrast, 4 of the 5 least significant pairs (p-val > 0.01) included the JHU study involving a single unusual tumor with neighboring small cell and adenocarcinoma foci sharing clonal mutations. We remark that the pairwise Fisher test provided only a rough comparison of studies, since the corresponding gene-sets depended on statistical details and differential expression methods.

Supp Table 3: To investigate the effect of primary versus metastatic tumors in the context of NEPC gene-lists (WCMC 2011 and VPC 2012), we assessed differential expression in 1000 sets of samples randomly drawn from the MSKCC dataset profiling 19 metastatic and 119 primary adenocarcinomas (of Gleason scores 5 (1), 6 (77), 7 (42), 8 (7), and 9 (4)). The random subset selections were constrained to have similar composition to the WCMC 2011 cohort, specifically 6 metastases versus 30 primaries of Gleason scores 5 (1), 6 (1), 7 (23), 8 (4), 9 (1); we did not account for the one primary NEPC in the WCMC 2011 cohort, although considered including a high grade primary into the metastases group to model its effect. Running LIMMA on these 1000 sample sets produced a median of 907 and 711.5 genes (out of 26446 total with quartiles 627.75-1369.5 and 554.75-933.25) that were differentially up and down expressed in the metastatic adenocarcinomas at an adjusted p-value cutoff of 0.05. In terms of the statistically strongest genes, there were 30 and 80 genes that were consistently differentially up and down expressed in 95% or more of the 1000 comparisons. Of these, 17 (57%) and 7 (9%) were in the WCMC 2011 gene-list, thus confirming that the WCMC 2011 up gene-list in particular was enriched for metastases-related up genes (Fisher p=5.5e-19). Similarly, the VPC 2012 up gene-list also had significant overlap with these metastases-related up genes (Fisher p=5.2e-15). Under gene-ontology analysis, the 30 metastases-related up genes were most enriched for mitotic nuclear division (9 genes; adj p=7.9e-7).

Supp Table 4: List of 23 NEPC samples from 15 unique patients across 6 datasets used for meta-analysis. Nine of these patients were used in deriving 5/8 literature NEPC gene-lists. Corresponding adenocarcinomas were largely from similar clinical stages, although the VPC dataset had a greater proportion of NEPC patients with metastases (1/2) than adenocarcinoma patients with metastases (1/7). *UW mCRPC and MDA CRPC contained 5 and 2 samples from 2 and 1 patients classified as adenocarcinoma with neuroendocrine differentiation, which were not included in calculation of adenocarcinoma means and standard deviations in the outlier analysis. Notation: m - metastatic, l - localized, CR - castrate resistant, PC - prostate cancer.

Supp Table 5: Genes with outlier status in 60-80% of the 15 NEPC patients included known NEPC-related genes such as PEG10, EZH2, CHGA, CHGB, REST, and RB1. Cell-cycle and proliferation genes such as TOP2A often had this level of outlier expression due to meeting outlier criteria for NEPC samples from xenograft and primary datasets but not necessarily in the metastatic CRPC setting.

Supp Table 6: List of 385 differentially expressed genes from LIMMA satisfying adjusted p-value < 0.05 after Benjamini-Hochberg correction for ~24000 total genes. All SCPC (N=16) versus adenocarcinomas (N=16) were compared, yielding 111 differentially up-expressed genes in SCPC and 274 down-expressed genes.

Supp Table 7: Lists of differentially expressed genes from 3 LIMMA comparisons satisfying adjusted p-value < 0.05 after Benjamini-Hochberg correction for ~24000 total genes. List 1 compared 4 atypical SCPC versus 9 prototypical adenocarcinomas yielding 118 genes (116 up, down). List 2 compared 9 prototypical SCPC versus 4 atypical SCPC yielding 115 genes (1 up, 114 down). List 3 compared 9 prototypical SCPC versus 9 prototypical adenocarcinomas yielding 1624 genes (381 up, 1243 down). The 3-centroid-model was based on common genes (176) from 2 or more of these lists (Table 5).

Supplementary Methods

Bioinformatic processing and analysis of gene expression datasets: Raw microarray data was processed in R by RMA-based pipelines to arrive at absolute log-intensities by probe and then further summarized by gene based on gene assignments derived from cross-referencing exon chromosomal locations in GENCODE v19 with probe alignment locations from annotation files (NetAffx, GPL6480, GPL14550, GPL15659). For exon arrays, Aroma.Affymetrix or Affymetrix Power Tools were used. Exon-level processing of all ~1.4 million probe-sets was performed, followed by gene-summarization based on gene assignments and probe-set evidence level (core, extended, or full), with restriction to core probe-sets when available for a gene, to extended probe-sets when a gene had no core probe-sets, and to full probe-sets otherwise. A primary summarization was generated from an abbreviated set of ~24000 genes. Batch correction of exon or gene-level data was applied with the ComBat package to account for different RNA processing dates, and a covariate representing adenocarcinoma or small cell status was also used due to imbalanced batches [[5](#_ENREF_5)]. A recent study reported that covariates may potentially exaggerate differential expression, although our dataset had less differentially expressed genes than expected (see Results) [[6](#_ENREF_6)]. Two small cell samples (54674 and 57917) had outlier NUSE quality scores > 1.05, however were nonetheless included in downstream analysis since they clustered with other small cell samples under NE gene-sets.

For gene microarrays, single-channel arrays were processed by RMA for Affymetrix chips (GSE32967; R affyPLM), or by normexp background correction with offset 16 followed by quantile normalization between arrays for Agilent chips (GSE41192; R LIMMA). Two-channel Agilent microarrays underwent similar background correction and also Loess normalization within arrays, then absolute log-intensity values were generated according to steps outlined in [[7](#_ENREF_7)]. Specifically, if a common reference channel was used (GSE35988, GSE66187, LuCaP), then Gquantile normalization between arrays was performed to ensure the same empirical distribution across reference samples while keeping log-ratios unchanged, and values for study samples were produced by log-ratio offsets to the median of the normalized reference values. By contrast, if there were no common reference sample, so that every channel contained a study sample (GSE33277), then the channels within each individual array were first adjusted so that log-ratios agreed with Loess normalization but average log-intensity remained unchanged, followed by single-channel quantile normalization between arrays. Duplicate probes were averaged, and probes with the same gene assignment were averaged. Batch correction was performed for the UM dataset based on chip type and for the UW dataset based on labeled batch. For MDA xenografts, technical replicates were averaged, and for VPC xenografts, biological replicates were removed so that only one xenograft per clinical patient remained, except for the NEPC samples and castrate resistant pairs. For the MDA CRPC dataset, benign and xenograft samples were removed, and for the UM CRPC dataset, normal and primary samples were removed.

For RNA-seq data, processed data was obtained from cBioPortal, transformed by log2(x+1), and genes below 3 across all samples were excluded.

Datasets exhibited characteristic distributions based on platform. Variance of NEPC samples versus adenocarcinoma samples was calculated from processed data, and for datasets with multiple samples from the same patient, mean variance was calculated over all combinations with one sample per patient (or 1000 random combinations in the case of WCMC). LIMMA was used to identify differentially expressed genes at an adjusted p-value cut-off of 0.05 after Benjamini-Hochberg correction [[8](#_ENREF_8)]. GSEA was used to test enrichment, and DAVID and PANTHER were used for gene-ontology analysis of biological processes [[9-11](#_ENREF_9)].

Low AR signaling and low neuronal phenotype analysis: We identified samples with outlier low ARS scores (fold-change < -1, z-score < -2) in clinical datasets: MDA, UM CRPC, UW CRPC, SU2C, WCMC, JHU-FFPE, MSKCC. In the JHU-FFPE and WCMC, outlier criteria were slightly relaxed due to attenuated expression (fold-change < -0.75, z-score < -1.5). Among outlier samples, we then identified those with low or regular neuronal phenotype expression by using moderate cutoffs relative to this gene signature (fold-change < 0.5, z-score < 1), which excluded most NEPC samples. We then performed 2 separate LIMMA-based differential expression analyses comparing the remaining samples first with NEPC and second with adenocarcinoma samples. Since some datasets had only 1 or 2 of these samples, we merged all gene expression data together on the level of fold-changes relative to adenocarcinomas. Differentially expressed genes common to the 2 LIMMA analyses were then ordered by average rank.

Bibliography

1. Kumar, A., et al., *Substantial interindividual and limited intraindividual genomic diversity among tumors from men with metastatic prostate cancer.* Nat Med, 2016. 22(4): p. 369-78.

2. Beltran, H., et al., *Molecular characterization of neuroendocrine prostate cancer and identification of new drug targets.* Cancer Discov, 2011. 1(6): p. 487-95.

3. Beltran, H., et al., *Divergent clonal evolution of castration-resistant neuroendocrine prostate cancer.* Nat Med, 2016. 22(3): p. 298-305.

4. Rajan, P., et al., *Next-generation sequencing of advanced prostate cancer treated with androgen-deprivation therapy.* Eur Urol, 2014. 66(1): p. 32-9.

5. Johnson, W.E., C. Li, and A. Rabinovic, *Adjusting batch effects in microarray expression data using empirical Bayes methods.* Biostatistics, 2007. 8(1): p. 118-27.

6. Nygaard, V., E.A. Rodland, and E. Hovig, *Methods that remove batch effects while retaining group differences may lead to exaggerated confidence in downstream analyses.* Biostatistics, 2016. 17(1): p. 29-39.

7. Yang, Y.H. and P.T. Natalie, *Normalization for Two-Color cDNA Microarray Data.* Lecture Notes-Monograph Series, 2003. 40: p. 403-418.

8. Ritchie, M.E., et al., *limma powers differential expression analyses for RNA-sequencing and microarray studies.* Nucleic Acids Res, 2015. 43(7): p. e47.

9. Subramanian, A., et al., *Gene set enrichment analysis: a knowledge-based approach for interpreting genome-wide expression profiles.* Proc Natl Acad Sci U S A, 2005. 102(43): p. 15545-50.

10. Huang da, W., B.T. Sherman, and R.A. Lempicki, *Systematic and integrative analysis of large gene lists using DAVID bioinformatics resources.* Nat Protoc, 2009. 4(1): p. 44-57.

11. Thomas, P.D., et al., *PANTHER: a library of protein families and subfamilies indexed by function.* Genome Res, 2003. 13(9): p. 2129-41.
