# Supplementary material for: Gene expression signatures of neuroendocrine prostate cancer and primary small cell prostatic carcinoma
Source: BMC Cancer. 2017 Nov 13;17:759. doi: 10.1186/s12885-017-3729-z (PMC5683385; doi:10.1186/s12885-017-3729-z)
Supplement: Supplementary file 3 — Additional figures on meta-12 scores, AR signaling versus AR / CCP / RAB3B, mixed tumors, REST exons, batch effects, principal components, and correlation strengths. (PDF 13484 kb) [file 12885_2017_3729_MOESM3_ESM.pdf]

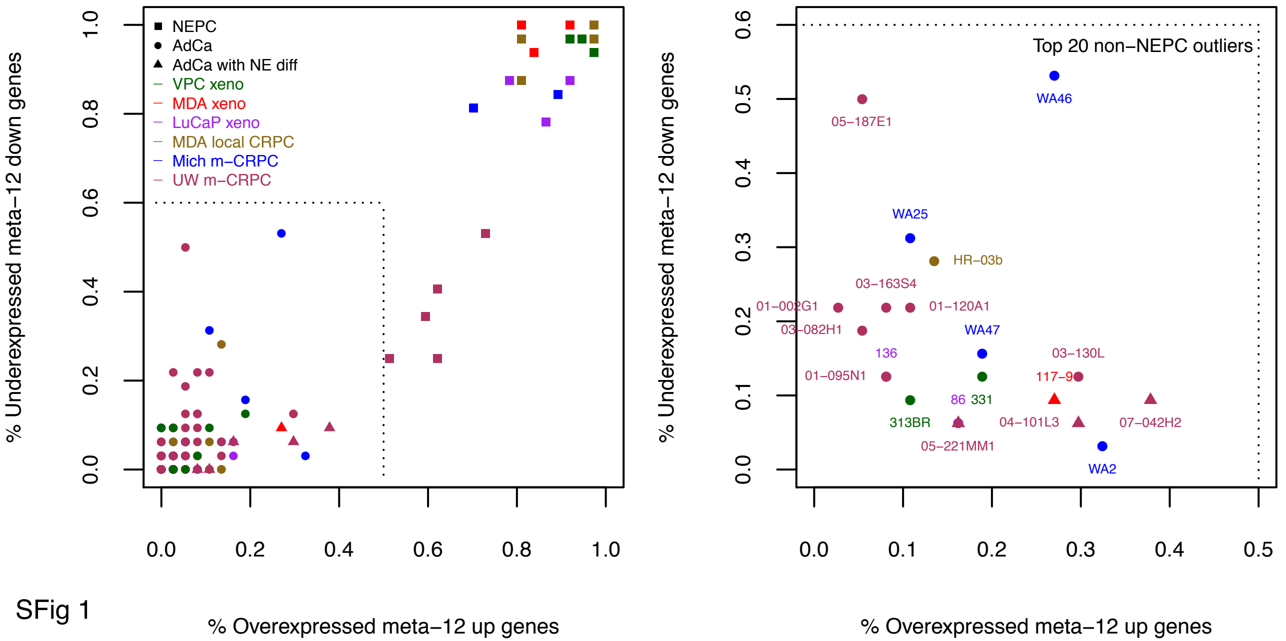

NEPC
AdCa
AdCa with NE diff
VPC xeno
MDA xeno
LuCaP xeno
MDA local CRPC
Mich m–CRPC
UW m–CRPC
% Underexpressed meta–12 down genes
% Overexpressed meta–12 up genes
0.0
0.2
0.4
0.6
0.8
1.0
Top 20 non–NEPC outliers
WA46
05–187E1
WA25
HR–03b
03–163S4
01–002G1
01–120A1
03–082H1
WA47
136
03–130L
01–095N1
117–9
86
331
313BR
04–101L3
07–042H2
05–221MM1
WA2
0.1
0.3
0.5

SFig 1

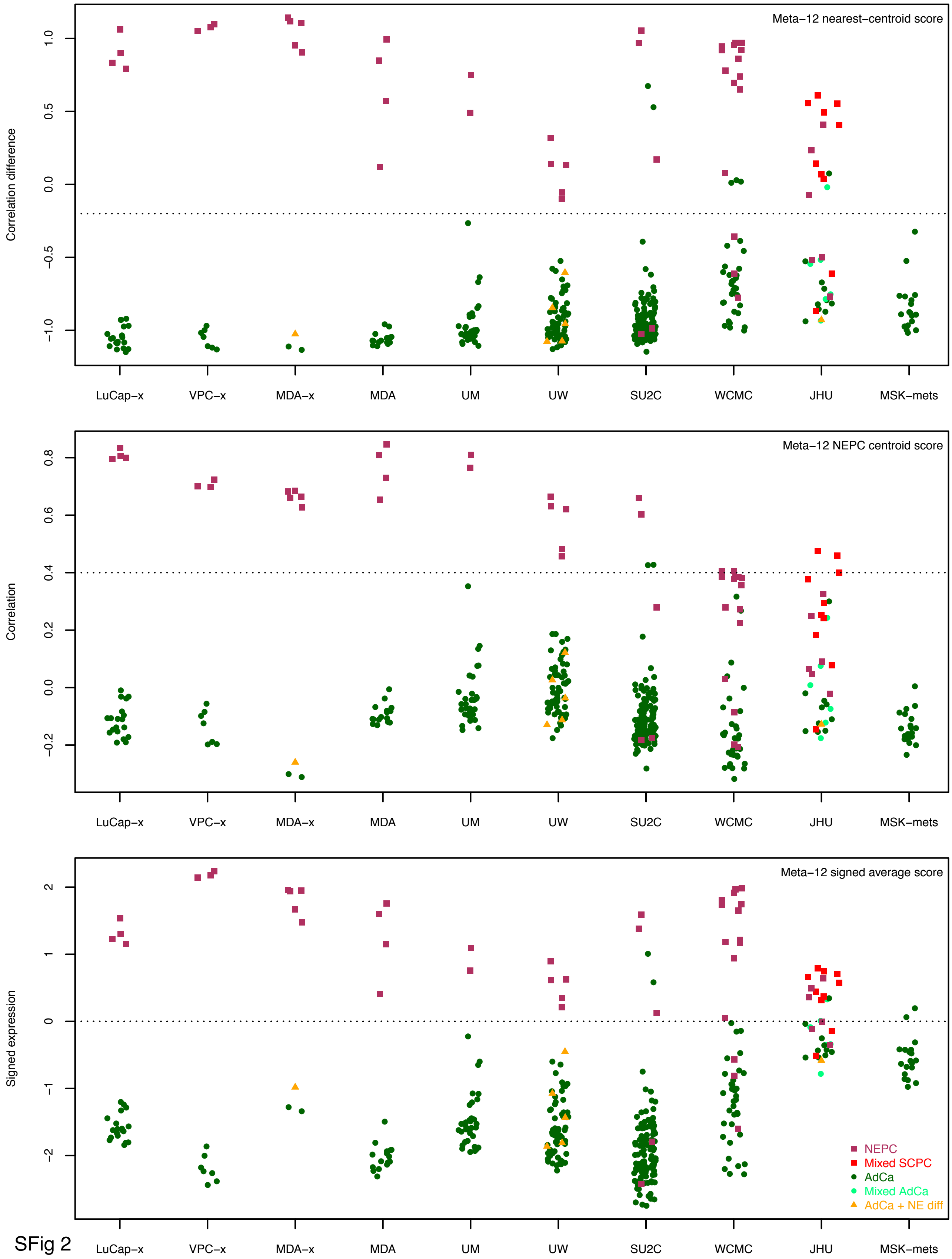
Meta−12 nearest−centroid score
Correlation difference
1.0
0.5
0.0
−0.5
−1.0
Meta−12 NEPC centroid score
Correlation
0.8
0.6
0.4
0.2
0.0
−0.2
Meta−12 signed average score
Signed expression
2
1
0
−1
−2
LuCap−x
VPC−x
MDA−x
MDA
UM
UW
SU2C
WCMC
JHU
MSK−mets
NEPC
Mixed SCPC
AdCa
Mixed AdCa
AdCa + NE diff

SFig 2

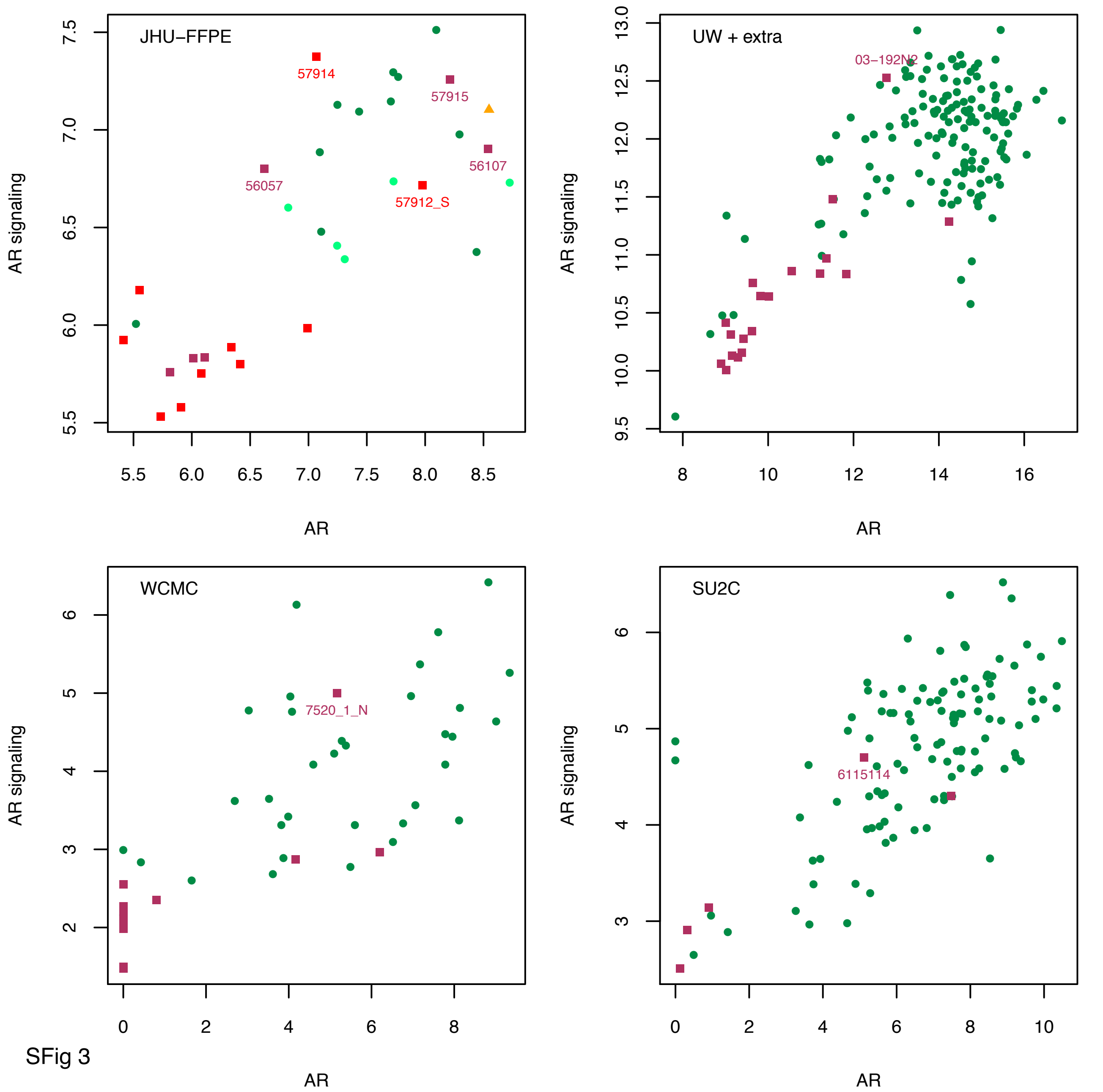
JHU−FFPE
57914
57915
56107
56057
57912_S
UW + extra
03−192N2
WCMC
7520_1_N
SU2C
6115114
AR signaling
AR

SFig 3

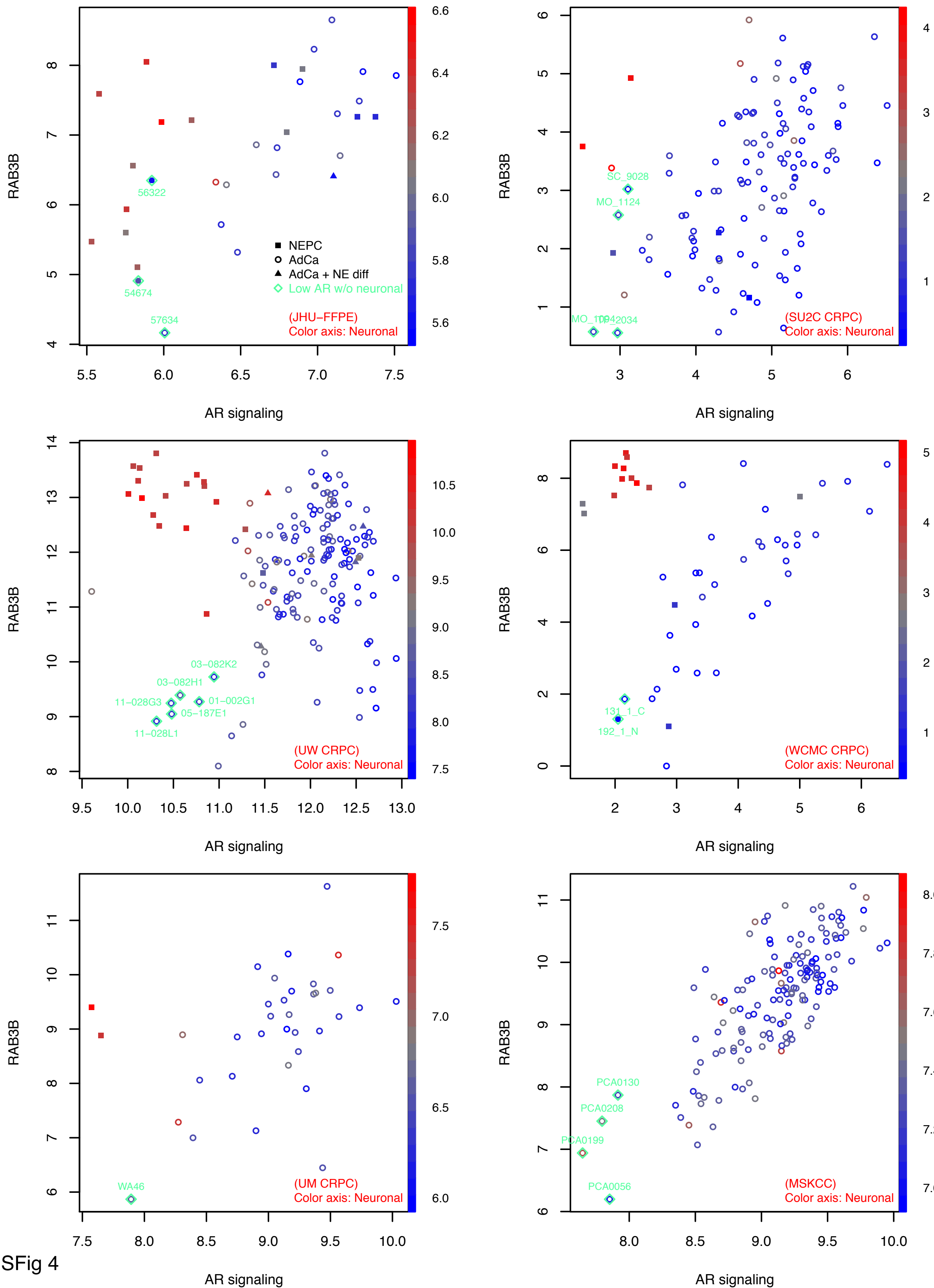
RAB3B
AR signaling
NEPC
AdCa
AdCa + NE diff
Low AR w/o neuronal
56322
54674
57634
(JHU–FFPE)
Color axis: Neuronal
SC_9028
MO_1124
MO_1094
2034
(SU2C CRPC)
Color axis: Neuronal
03–082K2
03–082H1
11–028G3
01–002G1
05–187E1
11–028L1
(UW CRPC)
Color axis: Neuronal
131_1_C
192_1_N
(WCMC CRPC)
Color axis: Neuronal
WA46
(UM CRPC)
Color axis: Neuronal
PCA0130
PCA0208
PCA0199
PCA0056
(MSKCC)
Color axis: Neuronal

SFig 4

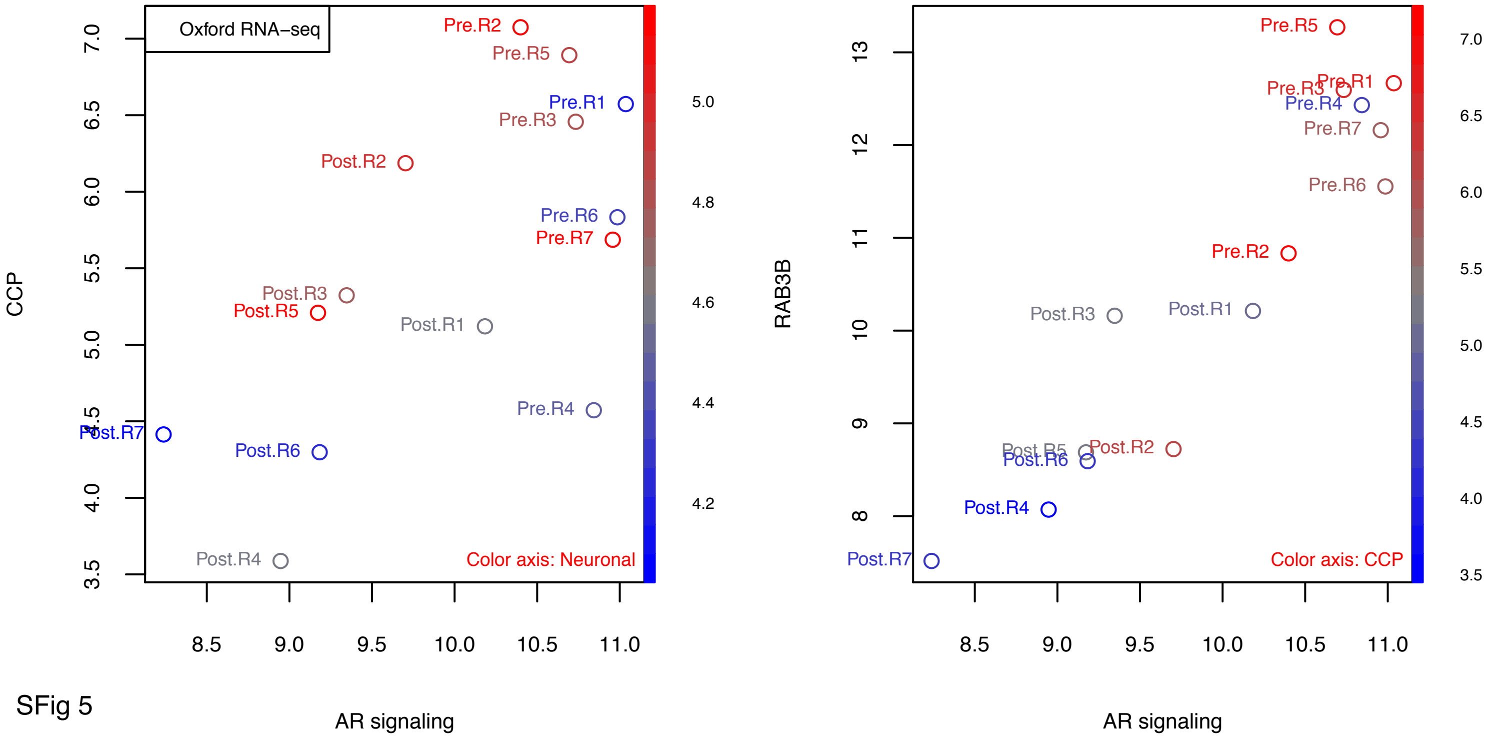
Oxford RNA−seq
CCP
AR signaling
Color axis: Neuronal
Pre.R2
Pre.R5
Pre.R1
Pre.R3
Post.R2
Pre.R6
Pre.R7
Post.R3
Post.R5
Post.R1
Pre.R4
Post.R7
Post.R6
Post.R4
RAB3B
AR signaling
Color axis: CCP
Pre.R5
Pre.R1
Pre.R3
Pre.R4
Pre.R7
Pre.R6
Pre.R2
Post.R3
Post.R1
Post.R5
Post.R2
Post.R6
Post.R4
Post.R7

SFig 5

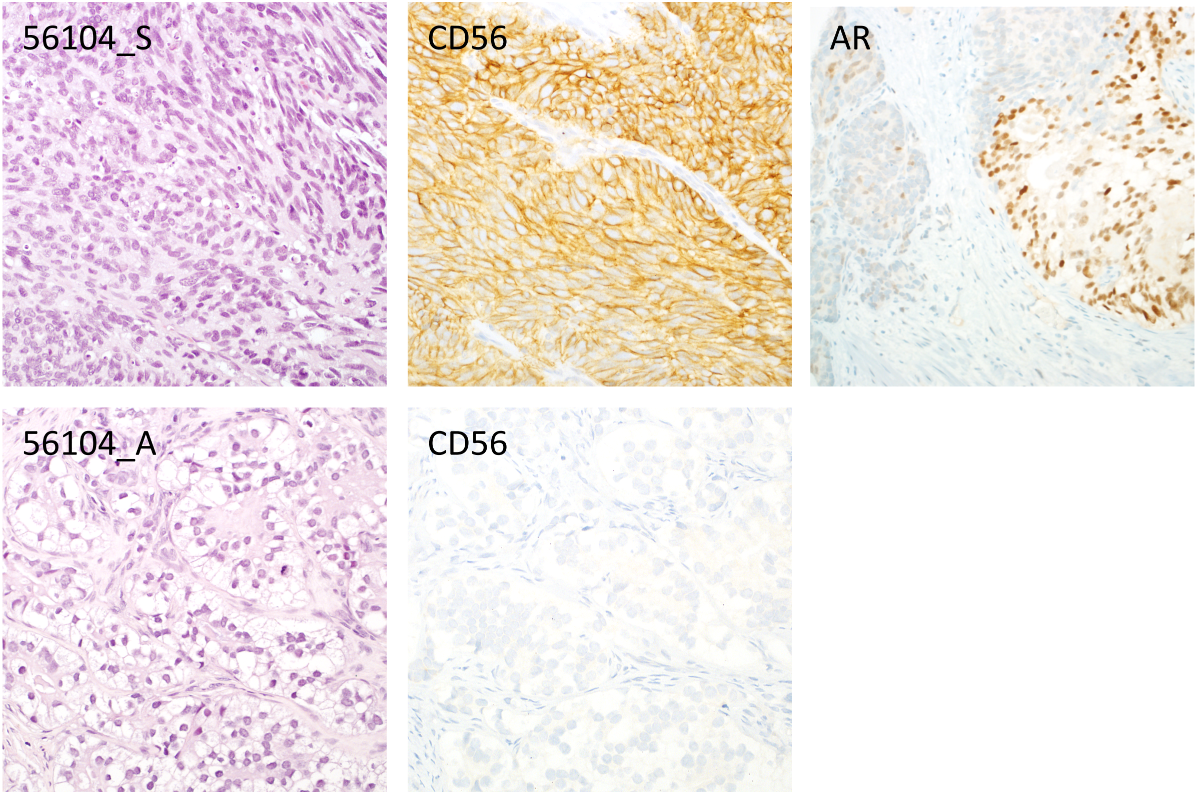

SFig 6

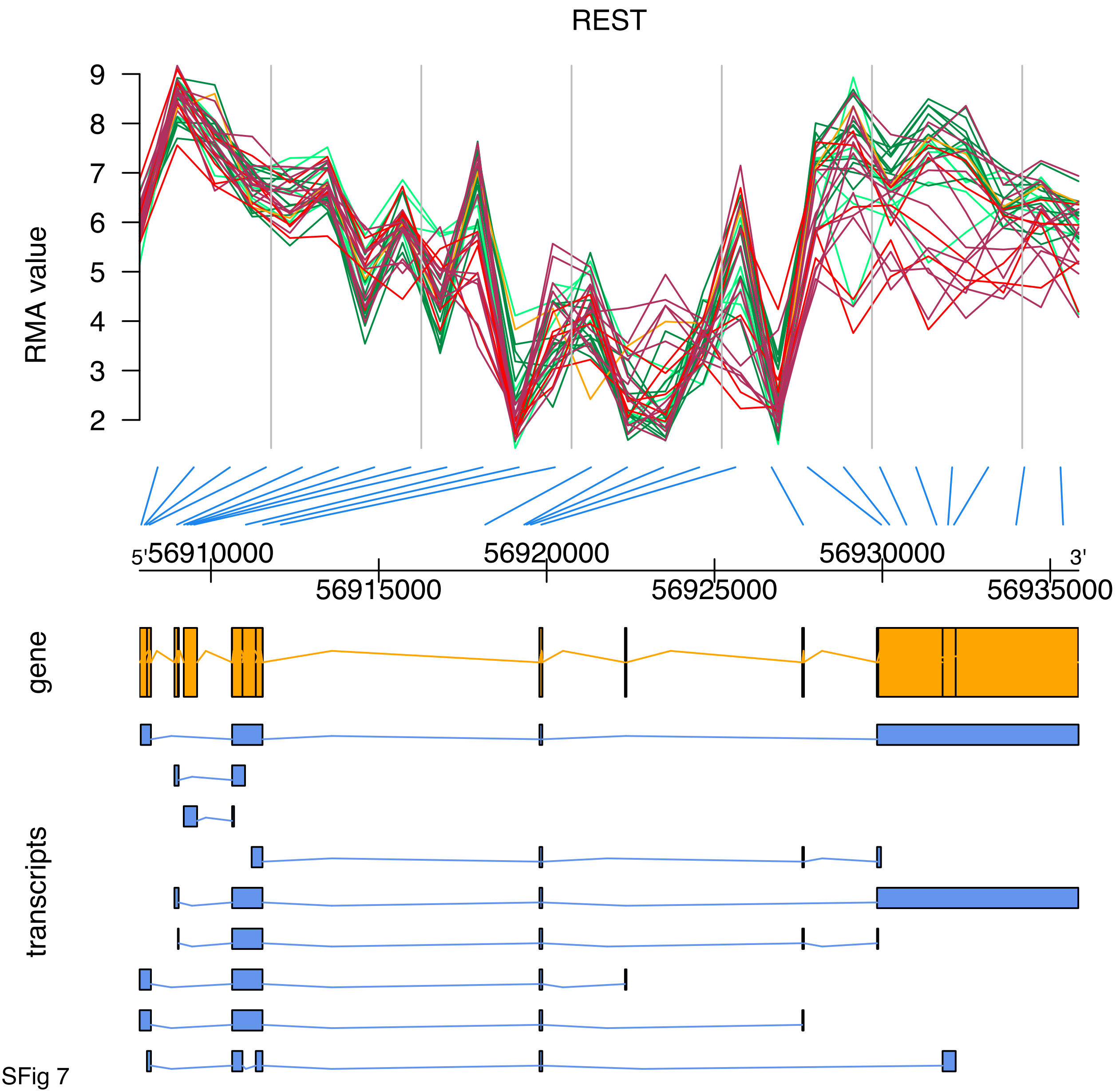
REST
RMA value
9
8
7
6
5
4
3
2
5'
56910000
56920000
56930000
3'
56915000
56925000
56935000
gene
transcripts

SFig 7

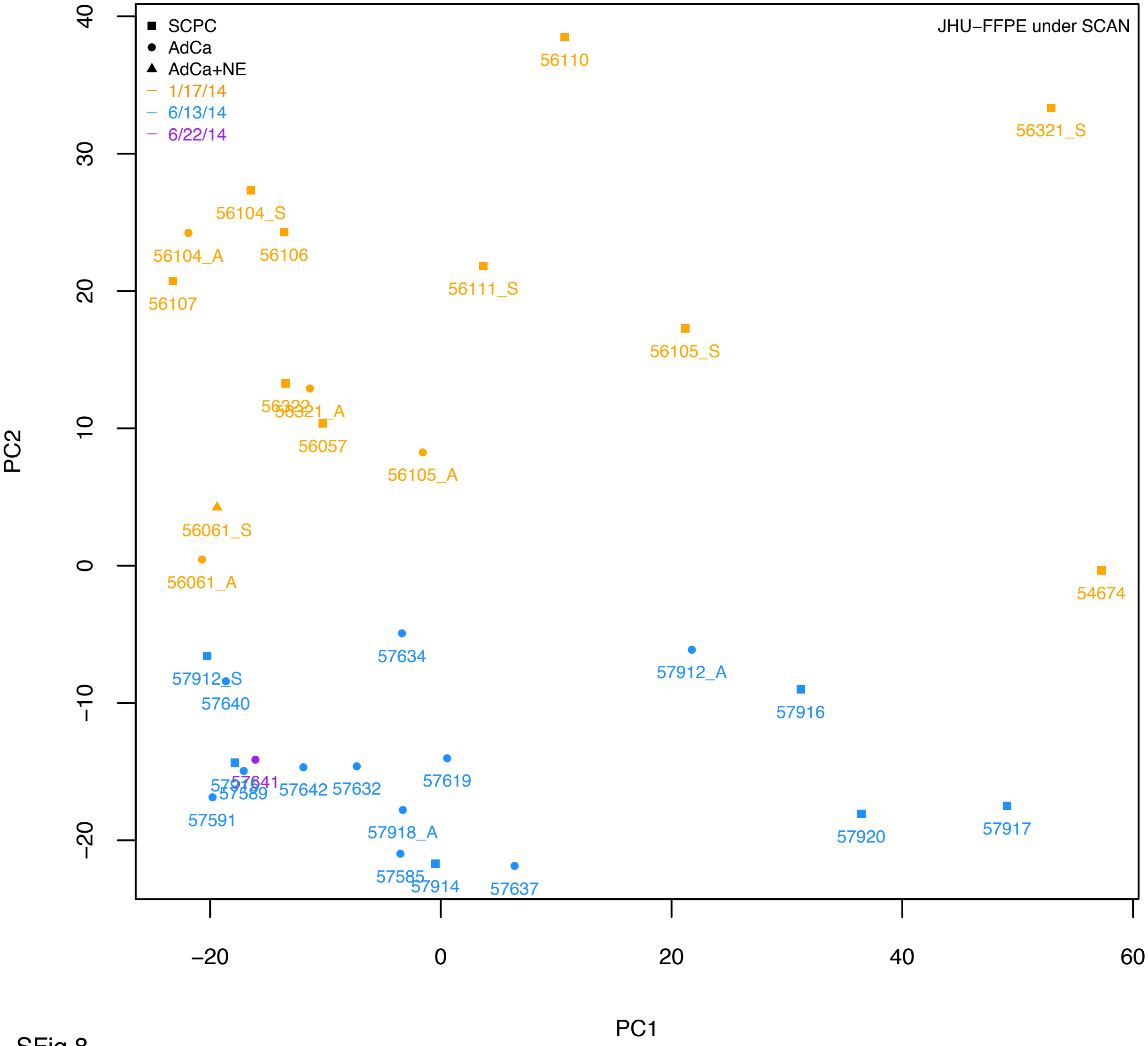

SCPC
AdCa
AdCa+NE
1/17/14
6/13/14
6/22/14
JHU−FFPE under SCAN
56110
56321_S
56104_S
56104_A
56106
56107
56111_S
56105_S
56322
56321_A
56057
56105_A
56061_S
56061_A
54674
57634
57912_S
57640
57912_A
57916
57916
57641
57589
57642
57632
57619
57591
57918_A
57920
57917
57585
57914
57637
PC1
PC2

SFig 8

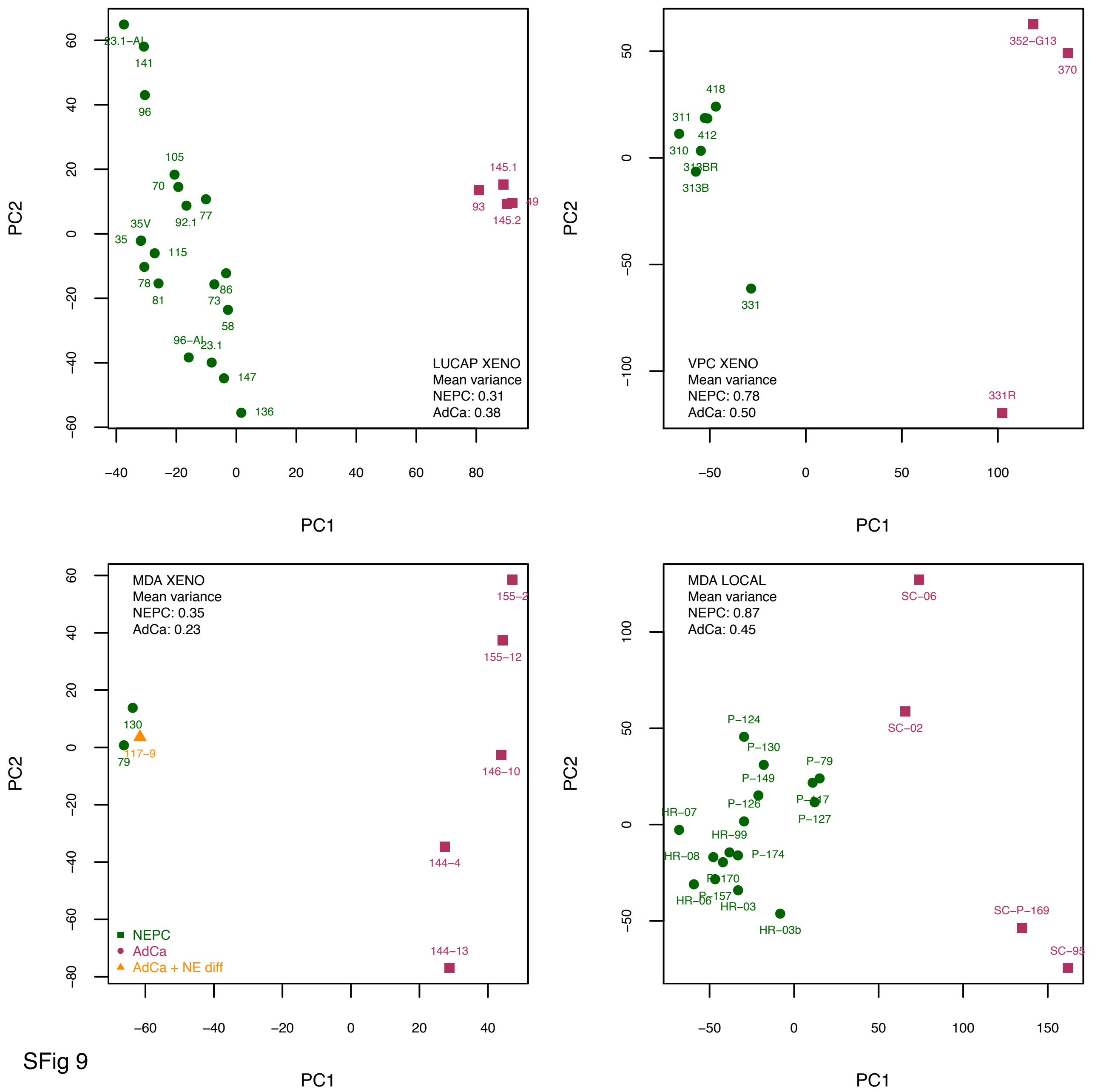
LUCAP XENO
Mean variance
NEPC: 0.31
AdCa: 0.38
VPC XENO
Mean variance
NEPC: 0.78
AdCa: 0.50
MDA XENO
Mean variance
NEPC: 0.35
AdCa: 0.23
MDA LOCAL
Mean variance
NEPC: 0.87
AdCa: 0.45
NEPC
AdCa
AdCa + NE diff
PC1
PC2

SFig 9

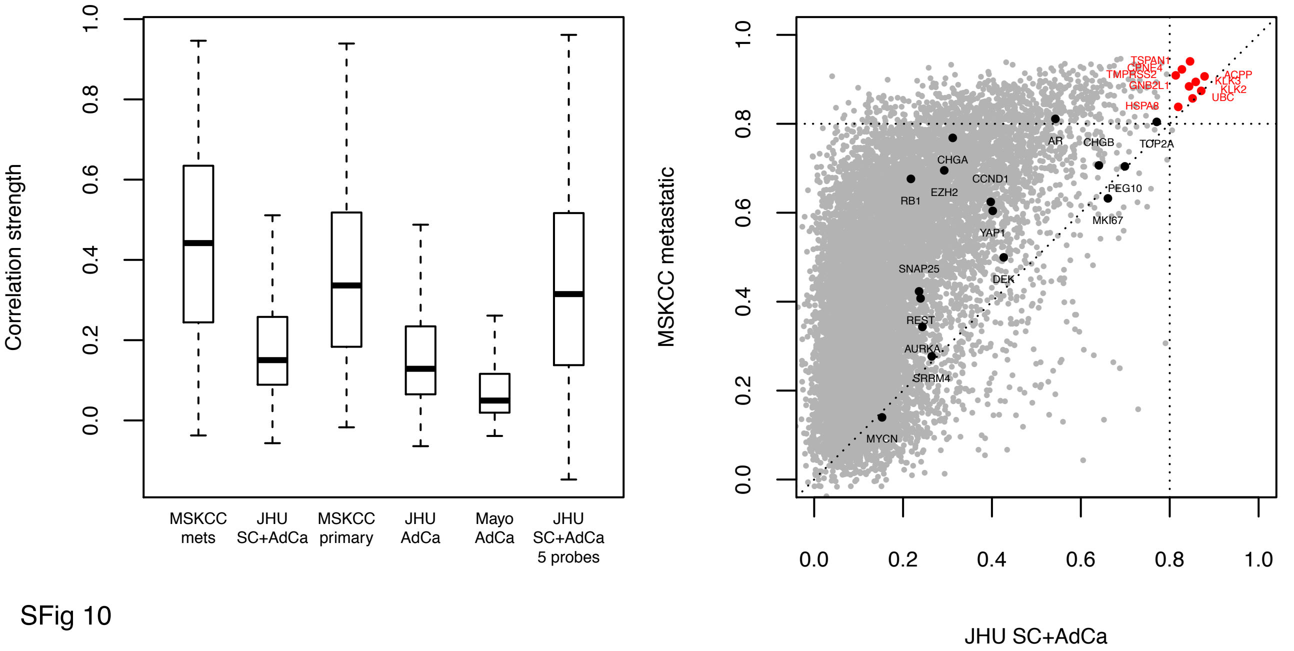

Correlation strength
0.0
0.2
0.4
0.6
0.8
1.0
MSKCC mets
JHU SC+AdCa
MSKCC primary
JHU AdCa
Mayo AdCa
JHU SC+AdCa 5 probes
MSKCC metastatic
JHU SC+AdCa
TSPAN1
CPNE4
TMPRSS2
GNB2L1
HSPA8
ACPP
KLK3
KLK2
UBC
AR
CHGB
TOP2A
CHGA
CCND1
EZH2
RB1
PEG10
MKI67
YAP1
SNAP25
DEK
REST
AURKA
SRRM4
MYCN

SFig 10

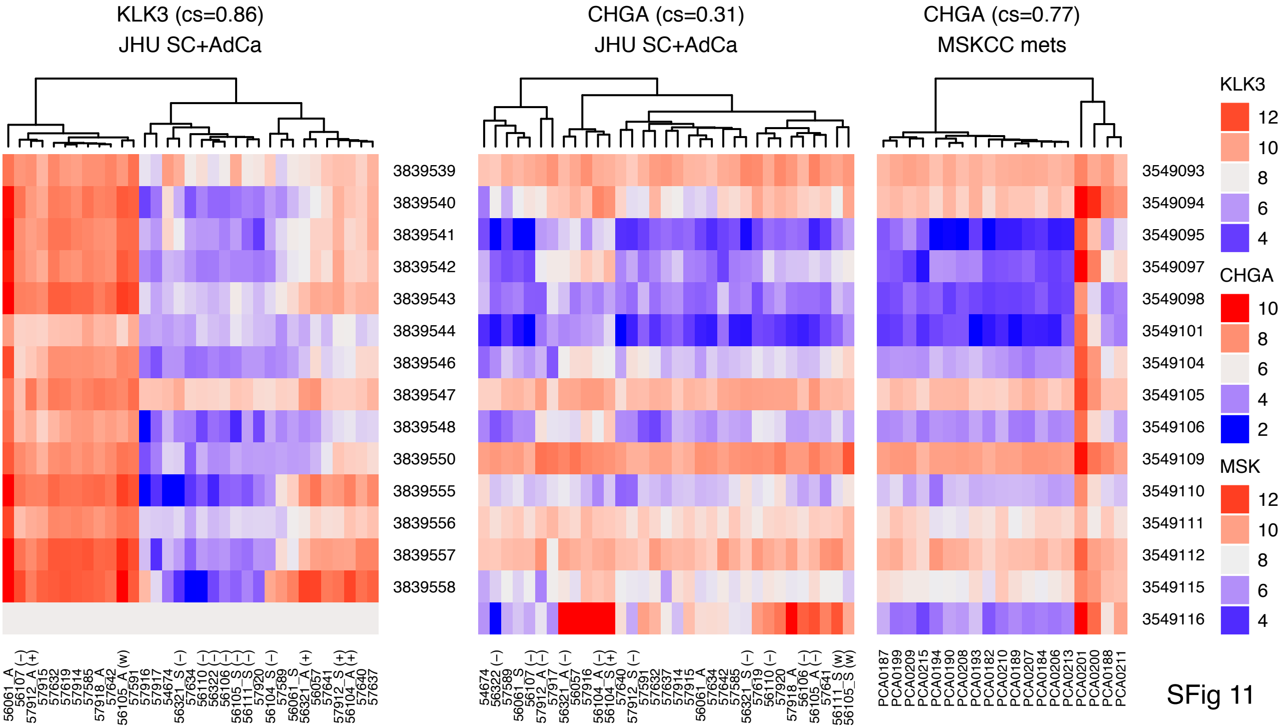
KLK3 (cs=0.86)
JHU SC+AdCa
CHGA (cs=0.31)
JHU SC+AdCa
CHGA (cs=0.77)
MSKCC mets
3839539
3839540
3839541
3839542
3839543
3839544
3839546
3839547
3839548
3839550
3839555
3839556
3839557
3839558
3549093
3549094
3549095
3549097
3549098
3549101
3549104
3549105
3549106
3549109
3549110
3549111
3549112
3549115
3549116
KLK3
12
10
8
6
4
CHGA
10
8
6
4
2
MSK
12
10
8
6
4
56061_A
56107_(-)
57912_A_(+)
57915
57632
57619
57914
57585
57918_A
57642
56105_A_(w)
57591
57916
57917
54674
56321_S_(-)
57634
56110_(-)
56322_(-)
56106_(-)
56105_S_(-)
56111_S_(-)
57920
56104_S_(-)
57589
56061_S
56321_A_(+)
56057
57641
57912_S_(+)
56104_A_(+)
57640
57637
54674
56322_(-)
57589
56061_S
56107_(-)
57912_A_(-)
57917
56321_A_(-)
56057
57916
56104_A_(-)
56104_S_(+)
57640
57912_S_(-)
57591
57632
57637
57914
57915
56061_A
57634
57642
57585
56321_S_(-)
57619
56110_(-)
57920
57918_A
56106_(-)
56105_A_(-)
57641
56111_S_(w)
56105_S_(w)
PCA0187
PCA0199
PCA0209
PCA0215
PCA0194
PCA0190
PCA0208
PCA0193
PCA0182
PCA0210
PCA0189
PCA0207
PCA0184
PCA0206
PCA0213
PCA0201
PCA0200
PCA0188
PCA0211

SFig 11
